# Silver-Loaded *Turbinaria turbinata* Oil Nanoemulsions: Antimicrobial and Anticancer Potential Revealed Through In Vitro Assays and Molecular Docking

**DOI:** 10.3390/md24070244

**Published:** 2026-07-13

**Authors:** Ragaa A. Hamouda, Abrar M. Alhumairi, Roaa M. Alreemi

**Affiliations:** 1 Department of Applied Radiologic Technology, College of Applied Medical Sciences, University of Jeddah, Jeddah 23218, Saudi Arabia; 2 Faculty of Biotechnology, University of Sadat City, Sadat City 32897, Egypt; 3 Department of Biological Sciences, Applied College at Khulais, University of Jeddah, Jeddah 21959, Saudi Arabia; amhumairi@uj.edu.sa; 4 Department of Biological Sciences, College of Science, University of Jeddah, Jeddah 21956, Saudi Arabia; rmalreemi@uj.edu.sa

**Keywords:** nanoemulsions, silver, brown algae, anticancer, antibacterial, molecular docking

## Abstract

Nanoemulsions are promising nanotechnology-based delivery systems that may improve the stability, bioavailability, and cellular uptake of therapeutic agents. Silver nanoparticles (AgNPs) have been reported to exhibit high antibacterial and anticancer activities via several mechanisms, such as the generation of oxidative stress and disruption of cellular membrane integrity. Breast cancer (MCF−7) and ovarian cancer (SK-OV−3) represent two highly aggressive malignancies that pose major global health challenges. Brown algae oil is a natural marine-derived product with a number of bioactive compounds, including fatty acids, sterols, and antioxidants, responsible for its numerous biological activities. Oil extracted from the brown alga *Turbinaria turbinata*, using hexane as an organic solvent, was formulated with silver nitrate (AgNO_3_) using a surfactant-stabilized spontaneous emulsification method to prepare a silver-loaded *T. turbinata* oil nanoemulsion (Ag-TTO-NE). The biological performance of the system was evaluated against human cancer cell lines, including MCF−7 (breast cancer) and SK-OV−3 (ovarian cancer), in addition to pathogenic bacterial strains, and for antioxidant activity. The results demonstrated that the silver-loaded oil nanoemulsion (Ag-TTO-NE) exhibited anticancer activities against MCF−7 (breast cancer) and SK-OV−3, with IC50 values of 105.86 and 72.45 µg/mL and a Selectivity Index of 2.34 and 3.41, respectively. The silver-loaded oil nanoemulsion (Ag-TTO-NE) possessed antioxidant and antimicrobial activities against *Bacillus subtilis* (ATCC 6633), *Staphylococcus aureus* (ATCC 6538), *Pseudomonas aeruginosa* (ATCC90274) and *Salmonella typhi* (ATCC 6539). These results indicate that *T. turbinata*-based silver nanoemulsions deserve further exploration as multifunctional marine-derived nanoformulations. In silico ADMET analysis projected moderate to high oral absorption for most of the discovered compounds and suggested favorable pharmacokinetic properties of the individual ingredients. ADMET analysis suggested that the major compounds discovered by GC–MS have good medication-like characteristics. These computational predictions are supplemental information and are not to be taken as the pharmacokinetic behavior of the nanoemulsion itself. Overall, the present results are based on in vitro biological assays together with exploratory computational studies and constitute preliminary evidence for the subsequent exploration of this marine-derived nanoformulation.

## 1. Introduction

Breast cancer is one of the most common cancers among women worldwide and remains one of the most prevalent malignancies, with substantial morbidity and mortality [1]. Breast cancer continues to be the most frequently diagnosed disease and leading cause of cancer-related deaths among women globally. In 2018, an estimated two million new cases were diagnosed worldwide, predominantly commonly in post-menopausal women, and a significant proportion of these cases resulted in death. Estrogen receptor (ER)-positive breast cancers, in which estrogens stimulate tumor cell proliferation, account for approximately 70–80% of cases, and the estrogen receptor (ER) pathway is a prominent therapeutic target in breast cancer management [2]. Epithelial ovarian carcinoma represents the most prevalent subtype of ovarian cancer. The risk of developing this malignancy increases with age and is associated with several factors, including a family history of ovarian cancer, hereditary cancer syndromes, and mutations in the breast cancer susceptibility genes (BRCA). Furthermore, the incidence of ovarian cancer has shown a substantial increase among younger females [3]. In addition, ovarian cancer affects more than 300,000 women worldwide each year. It is the sixth most frequent malignancy in women and the fifth most common cause of cancer death in the UK. Most ovarian cancers are epithelial ovarian cancers, accounting for over 90% of cases, with additional types including germ cell tumors and stromal tumors [4].

Marine oils obtained from marine organisms are increasingly studied as sources of bioactive substances, particularly polyunsaturated fatty acids (PUFAs) such as omega−3 and omega−6 fatty acids. These functional oils exhibit multiple physiological benefits, including anti-inflammatory properties, obesity prevention, cardiovascular protection, neuroprotection, and possible anticancer effects [5]. Marine algae are considered a rich and sustainable source of bioactive metabolites such as carotenoids, sulfated polysaccharides, sterols, fatty acids, and phenolic compounds, many of which exhibit potential anticancer effects. These metabolites have been shown to exert anticancer activity via many mechanisms, including the induction of apoptosis, cell-cycle arrest, the modulation of oxidative stress, the inhibition of angiogenesis and the regulation of signaling pathways implicated in tumor progression [6]. Bioactive metabolites, including fatty acids, sterols, and phenolic compounds, have been extracted from brown macroalgae such as *Sargassum filipendula* and have been associated with antioxidant, antibacterial, and anticancer activity against various malignant cell lines [7]. Such biological actions have been related to several types of chemicals, such as carotenoids, sterols, terpenoids, polyphenols, and lipid-derived metabolites [8].

Marine algae have emerged as promising bioprospecting resources to produce sustainable nanotechnology, offering an environmentally friendly alternative to conventional chemical synthesis methods. The field of Phyconanotechnology has demonstrated that algae have the unique potential for the biosynthesis of metallic nanoparticles due to the action of secondary metabolites including polyphenols, polysaccharides, proteins, and enzymes, which act as both reducing and capping agents [9]. Recent studies have shown that different taxonomic groups of marine algae, including Phaeophyta (brown algae), Rhodophyta (red algae), and Chlorophyta (green algae), exhibit distinct bioactive profiles that influence both nanoparticle characteristics and their biological activities [10,11].

The concept of nanoemulsions represents an alternative to conventional single-component nanoparticle delivery systems by encapsulating nanoparticles within bioactive matrices for the expression of synergistic therapeutic activities. These formulations may overcome several limitations associated with conventional nanomedicine, including poor stability, limited therapeutic efficacy, and suboptimal biocompatibility [12]. Current research suggests algae-based nanoparticles exert their biological properties by the expression of more than one mode of action, such as the generation of reactive oxygen species, membrane lyses, DNA damage, and the induction of cell apoptosis, making them attractive candidates for antimicrobial and anticancer applications [11,13,14]. Despite these promising findings, the clinical translation of algae-based nanoparticles remains limited by several challenges. One of the major limitations is the lack of standardized protocols for nanoparticle synthesis, physicochemical characterization, and biological evaluation, which hinders comparisons among studies [15]. In addition, the chemical composition of algal extracts may vary considerably depending on the source and preparation conditions, thereby influencing nanoparticle characteristics and their biological activities, highlighting the need for rigorous quality control. Furthermore, most studies have focused on simple nanoparticle formulations, with limited attention given to the potential synergistic interactions among the diverse bioactive constituents present in algal extracts [16].

The field of nanoparticle synthesis from algae has witnessed remarkable growth, and algae have been established as excellent bioprospecting resources due their rich content of secondary metabolites, which serve as both reducing and capping agents during nanoparticle synthesis [9]. Comprehensive mechanistic studies have revealed that algal-mediated nanoparticle synthesis can occur through both intracellular and extracellular pathways, with the extracellular route being particularly attractive for biomedical applications because of its simpler purification process. Brown, red, and green algae exhibit distinctive bioactive profiles that influence nanoparticle characteristics and biological activities, providing diverse biosynthetic potential for the development of tailored therapeutic nanomaterials [17].

Recent studies indicate that algae-derived nanoparticles possess superior biocompatibility compared with chemically synthesized nanoparticles while maintaining considerable therapeutic potential. The movement towards the establishment of Phyconanotechnology has been driven by the understanding that algal production offers enhanced biocompatibility, reduced environmental damage, and inherent biological functionality compared with established chemical processes [18].

Nanoemulsions represent an advanced drug delivery system in which nanoparticles are incorporated into bioactive matrices to enhance therapeutic performance through the combined effects of multiple active constituents [12]. Unlike conventional single-component systems, nanoemulsions exploit the interactions among different bioactive components to improve therapeutic efficacy. Recent studies have demonstrated that nanoparticle-based delivery systems enhance cellular uptake, prolong circulation time, reduce systemic clearance, and enable controlled drug release [19,20,21]. The incorporation of algal oils into nanoparticle platforms takes advantage of the amphiphilic nature of algal lipids to improve dispersion stability and facilitate cellular uptake. Moreover, bioactive lipids, particularly polyunsaturated fatty acids, may provide additional therapeutic benefits beyond their stabilizing role and could contribute to synergistic biological effects when combined with algae-derived nanoparticles [22].

Recent studies have highlighted that silver nanoparticles (AgNPs) exert cytotoxic activity primarily by inducing excessive production of reactive oxygen species (ROS), leading to DNA damage and triggering programmed cell death (apoptosis) or necrosis [23].

Recent studies have identified the various mechanisms through which algae-derived nanoparticles exert antibacterial activities, including the generation of reactive oxygen species, membrane disruption, the inhibition of enzymes, and DNA damage [15]. Algotiml et al. [10], concluded that the silver nanoparticles from the algae species *Ulva rigida* possess strong antimicrobial efficacy against *Trichophyton mantigrophytes* (40 mm inhibition zone) and *Escherichia coli* (19 mm inhibition zone), with the minimum lethal concentration ranging from 32 to 64 μg/mL. Despite these promising findings, bacterial resistance to nanoparticles has also been reported, with proposed mechanisms including efflux pump overexpression, biofilm formation, and metabolic adaptation [23].

Current research has demonstrated the potential of algae-derived nanoparticles in cancer therapy through multiple mechanisms, such as DNA damage, mitochondrial disruption, and the induction of apoptosis [24]. The selectivity index (SI) is widely used to evaluate the preferential cytotoxicity of anticancer agents toward cancer cells relative to normal cells, and previous studies have reported SI values greater than 3.0 for appropriately designed algal nanoparticles, indicating favorable selectivity [16]. Hamouda et al. [25] demonstrated anticancer activity against cell lines for hepatocellular carcinoma, colon carcinoma, cervical carcinoma, and prostate carcinoma, with IC50 values of 50.46, 45.84, 78.42, and 68.12 μg/mL, respectively. The outcomes support the broad-spectrum anticancer capability of algae-derived nanomaterials but also the necessity for selectivity for therapeutic applications.

*Turbinaria turbinata* is a brown macroalga widely distributed in tropical and subtropical marine environments, including the Red Sea, where it represents an abundant and renewable marine biomass resource. Recent studies conducted along the Saudi Arabian Red Sea coast have revealed the potential of *T. turbinata* for integrated biotechnological applications such as biodiesel production and bioethanol generation, presenting environmental and economic values as a sustainable marine feedstock. The abundance along the Red Sea coast, ease of collecting, and high biomass availability make this species a promising candidate for future blue-economy and marine biorefinery applications [26].

Species of the genus *Turbinaria* are known to be good sources of industrially important bioactive chemicals such as sulfated polysaccharides, alginates, polyphenols, carotenoids, and lipid-derived metabolites [27]. The fucoxanthin present in *T. turbinata* and related species have antioxidant and health-promoting properties [28,29]. In addition, sulfated polysaccharides extracted from *T. turbinata* have been proposed as promising candidates for biotechnological and pharmaceutical applications [30]. Furthermore, the lipid fractions obtained from *Turbinaria* species contain saturated and unsaturated fatty acids that have attracted considerable attention for biofuel production [26].

It is important to note that the bioactive compounds extracted from brown macroalgae are strongly influenced by geographical location, harvesting season, seawater temperature, salinity, nutrient availability, irradiance, and other environmental circumstances. Significant variations may occur in lipids, fatty acids, fucoxanthin, phenolic compounds, and sulfated polysaccharides among populations collected from different regions or seasons [31]. *Turbinaria decurrens* contains numerous bioactive compounds with beneficial health effects and demonstrated cytotoxic activity [32]. Silver and gold nanoparticles synthesized by *T. ornata* exhibit significant antimicrobial efficacy against pathogenic microorganisms of medical importance [33]. Moreover, species of the genus *Turbinaria* have been reported to possess antioxidant, antimicrobial, anti-inflammatory, and antidiabetic characteristics that may be applicable within the pharmaceutical sector [34]. Although the number of research papers published on nanoemulsions and algae-based nanoparticles is increasing, there is a scarcity of information on the combined physicochemical characterization, biological evaluation, and computational analysis of silver-loaded nanoemulsions prepared from *Turbinaria turbinata* oil in one study. Furthermore, the correlation between the chemical composition of such marine-derived nanoformulations and their potential anticancer, antibacterial and antioxidant effects is still poorly studied. Thus, the present study was designed as a proof-of-concept investigation to establish a scientific foundation for the future development of *T. turbinata*-based multifunctional marine nanoformulations.

The aims of the present study were (1) to synthesize and characterize a silver-loaded *T. turbinata* oil nanoemulsion, (2) to evaluate its anticancer activity against MCF−7 and SKOV3 cell lines, (3) to assess its antibacterial and antioxidant activities, and (4) to perform molecular docking and ADMET analyses to predict its physicochemical, pharmacokinetic, and toxicity-related properties.

## 2. Results and Discussion

### 2.1. Nanoemulsion Characterization

#### 2.1.1. FTIR Analysis

**Figure 1:**
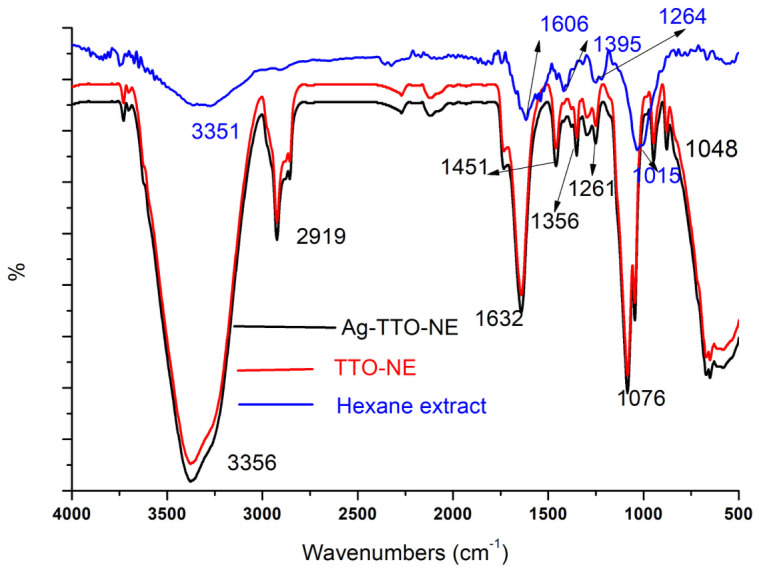
FTIR spectra analysis of the hexane extract, *T. turbinata* oil nanoemulsion (TTO-NE), and silver-loaded *T. turbinata* oil nanoemulsion (Ag-TTO-NE).

Figure 1 and Table 1 demonstrate the FTIR spectra of a silver-loaded *T. turbinata* alga-oil nanoemulsion (Ag-TTO-NE), *T. turbinata* alga-oil nanoemulsion (TTO-NE), and hexane extract of *T. turbinata*. FTIR analysis revealed no differences between Ag-TTO-NE and TTO-NE, whereas noticeable differences were observed compared with the hexane extract, indicating alterations in the chemical environment of several functional groups. The broad absorption band at 3356 cm^−1^ is characteristic of the O–H stretching vibrations associated with hydroxyl-containing compounds, including phenolics and alcohols [35].

The peak at 2919 cm^−1^ indicated the presence of aliphatic C–H stretching associated with long-chain hydrocarbons of fatty acids and lipid-based materials [35,36]. The absorption band at 1632 cm^−1^ may be attributed to the C=C stretching of unsaturated fatty acids and/or bending vibration of adsorbed water molecules, which are prevalent in biological systems including lipids [37,38]. The peaks at 1451 cm^−1^ and 1356 cm^−1^ correspond to deformation modes of CH_2_ and CH_3_ groups, respectively, indicating aliphatic hydrocarbon structures associated with lipid chains [36,37]. The band at 1261 cm ^−1^ corresponded to C–O stretching within ester functionalities, which is in good agreement with esterified fatty acid structures [39]. Peaks recorded at 1076 and 1048 cm^−1^ were consistent with stretching modes of C–O and C–O–C linkages, which could be due to ester functionalities, glycerol backbone vibrations or oxygenated surfactant-related groups derived from the surfactants used in the nanoemulsion formulation [35,37]. The FTIR spectrum generally indicated the dominance of hydroxyl, aliphatic hydrocarbon, ester carbonyl, and oxygen-containing functional groups, which are in accordance with the expected chemical composition of algal oil-based nanoemulsion systems.

#### 2.1.2. EDX Analysis

**Figure 2:**
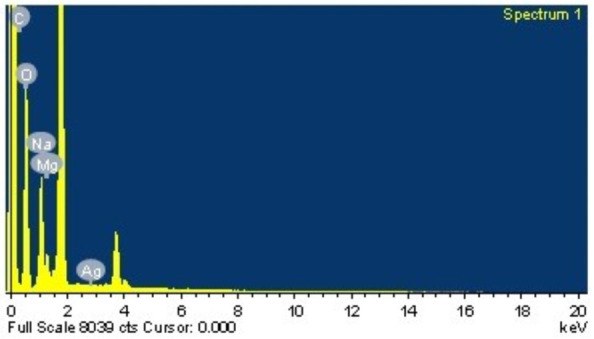
Energy dispersive X-ray spectroscopy analysis of silver-loaded *T. turbinata* oil nanoemulsion (Ag-TTO-NE).

The EDX elemental profile of the silver-loaded *T. turbinata* oil nanoemulsion (Ag-TTO-NE) (Figure 2 and Table 2) demonstrated the elemental composition of an organic–inorganic nanostructured system–bio-nanostructured system with unique organic–inorganic hybrid characteristics. The intense signals of carbon I and oxygen (O) in the low energy range (0–1 keV) are attributed to the rich biochemical content of the algal-derived matrix, primarily consisting of polysaccharides, proteins and lipid fractions. Figure 2 shows that the presence of mineral components in the algal biomass is supported by the presence of sodium (Na) and magnesium (Mg).

A characteristic silver signal was detected at ~3 keV, confirming the presence of silver within the nanoemulsion system of metallic silver in the oil nanoemulsion matrix. This spectral signature agrees with the usual emission of elemental Ag. The reduction process is probably mediated by redox active phytochemicals present in the algal extract, such as phenolic compounds, flavonoids, and reducing sugars, which have previously been reported to participate as natural electron donors. The same is true for the known biosynthetic pathways in alga-assisted nanoparticle manufacturing, where biomolecules contribute to both nanoparticle formation and nanoparticle stabilization, thereby reducing reliance on externally supplied chemical reagents [40]. The peak for silver was observed at 3 keV, in the olive oil nanoemulsion (NEM-olive oil) loaded with silver nanoparticles (Ag-NPs) derived from marine alga *T. turbinata* [21].

#### 2.1.3. Particle Size Distribution and Polydispersity Index (PDI)

**Figure 3:**
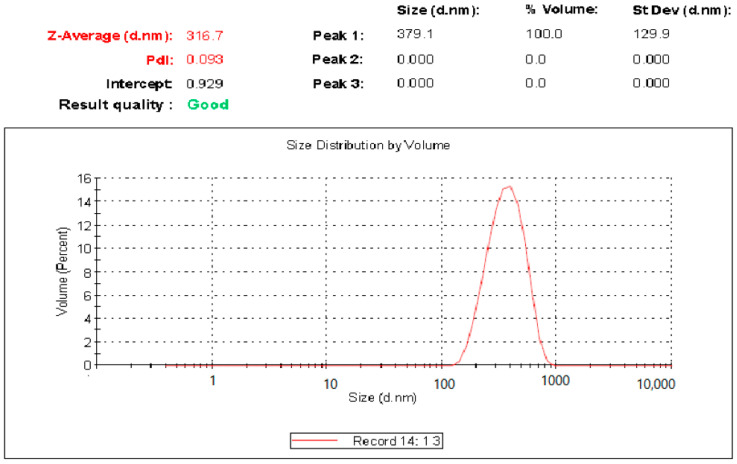
Zetasizer (DLS) measurement of silver-loaded *T. turbinata* oil nanoemulsion.

The dynamic light scattering (DLS) analysis of the silver-loaded *T. turbinata* oil nanoemulsion is shown in Figure 3, which reveals that the average hydrodynamic diameter of the Ag-algal oil nanoemulsion was 316.7 nm, with a low polydispersity index (PDI = 0.093). The low PDI value indicates a narrow particle size distribution and suggests a high degree homogeneity of the dispersed system. Generally, DLS-based characterization associates PDI values < 0.1 with very uniform colloidal dispersions, which means controlled particle formation and minimal particle aggregation [41].

The measured hydrodynamic diameter of 316.7 nm indicates that the designed system falls within the upper nanoscale to sub-micron region. The dimension is the hydrodynamic diameter, which includes the nanoparticle or emulsion core, the surrounding hydration layer, and the adsorbed biomolecular coating [42].

The combination of a relatively uniform particle size distribution and a very low PDI suggests favorable dispersion homogeneity under the tested conditions. Overall, the DLS results indicate that the prepared silver-loaded *T. turbinata* oil nanoemulsion possesses good dispersion uniformity and acceptable colloidal stability, supporting its suitability for biological and antimicrobial applications. This behavior may be associated with the fact that the separated polysaccharides and proteins from algae can induce surface aggregation and prevent the aggregation of particles during biosynthesis [43].

**Figure 4:**
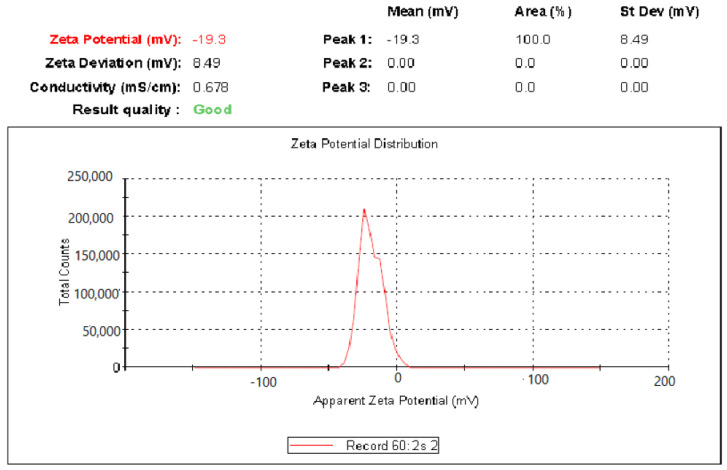
Zeta potential of the silver-loaded *T. turbinata* oil nanoemulsion.

The zeta potential of the synthesized silver-loaded *T. turbinata* oil nanoemulsion was measured to be −19.3 mV, which indicates that the dispersed particles possessed a moderate negative surface charge. The negative charge may be attributed to functional groups derived from algal biomolecules such as hydroxyl, carboxyl, and other oxygen-containing groups that are adsorbed onto the particle surface during biosynthesis (Figure 4).

This measured zeta potential suggests moderate electrostatic repulsion between the dispersed particles. In colloidal systems, the zeta potential values do not provide direct evidence of colloidal stability, because the stability of dispersion might be due to electrostatic repulsion as well as steric stabilization, especially in bio-mediated nanoparticle systems [42]. Comparable zeta potential values have been reported for other bio-based nanoemulsion systems; a tree oil nanoemulsion initiated zeta potential variations from −17.75 to −29.24 mV, a hyaluronic acid-based nanoemulsion formula exhibited a zeta potential of −23.9 mV, and an olive oil nanoemulsion exhibited (−19.8) [21].

#### 2.1.4. TEM Image Analysis

**Figure 5:**
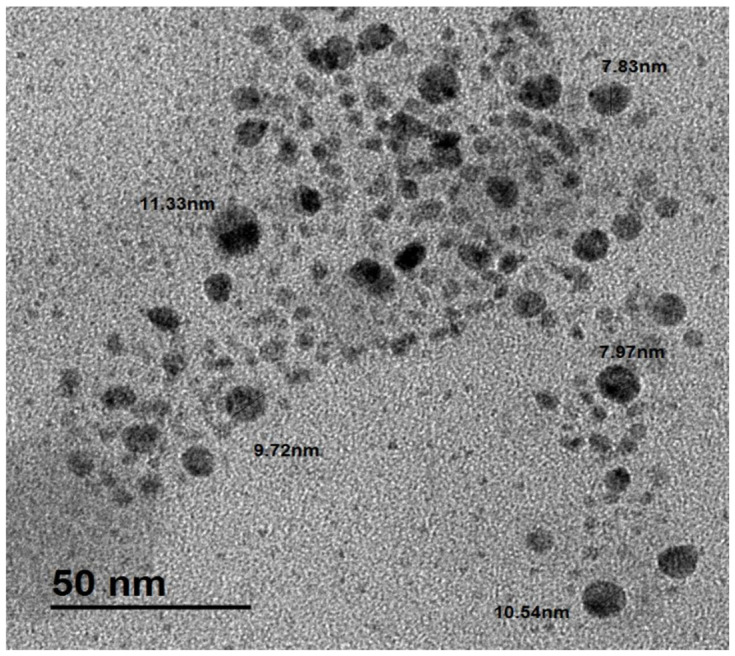
TEM images of synthesized silver-loaded *T. turbinata* oil nanoemulsion.

Figure 5 shows the particle size and shape of the silver-loaded *T. turbinata* oil nanoemulsion. TEM images confirmed the presence of nanosized particles embedded in the algal oil nanoemulsion matrix. The shape of the particles was predominantly spherical to quasi-spherical, with the size ranging from approximately 5 to 12 nm, which indicates the successful formation of nanoscale particles with moderate size variation. Most of the particles were well dispersed and individually distinguishable, and some limited localized clustering was observed in some regions, suggesting the partial proximity of particles rather than severe agglomeration. The small particle size observed by TEM is consistent with the controlled yielding of nanoparticles, which can be attributed to the stabilizing impact of algal biomolecules during biosynthesis [44]. The spherical morphology and nanoscale are the most prevalent features reported for biologically synthesized AgNPs and are often associated with favorable surface properties [45]. Polydisperse, spherical nanoparticles, with the size ranging from 3 to 10.2 nm, were obtained with the Ag/nanoemulsion—olive oil [21]. The combination of the low PDI (0.093), DLS size (316.7 nm), and TEM core size (~5–12 nm) shows that the data are consistent with relatively uniform dispersion, which suggests favorable colloidal properties under the tested conditions. The difference between the TEM and DLS values is expected, as DLS indicates the hydrodynamic diameter incorporating hydration and the biomolecular coating, whereas TEM measures only the physical core size [46].

### 2.2. Phytochemical Analysis

**Figure 6:**
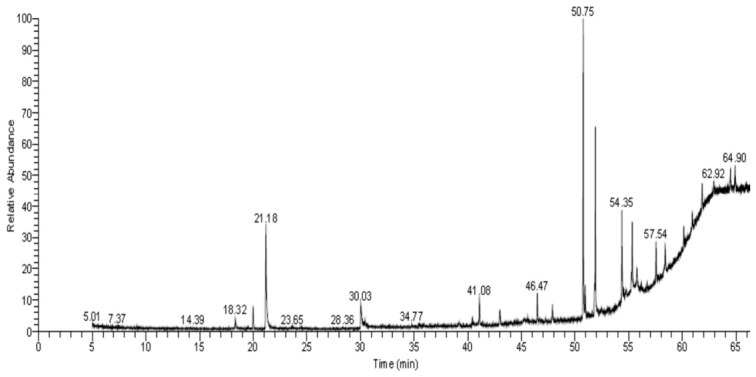
Representative GC–MS chromatogram of the Ag-loaded *T. turbinata* oil nanoemulsion (Ag-TTO-NE).

The GC–MS analysis of the silver-loaded *T. turbinata* oil nanoemulsion (Ag-TTO-NE) is depicted in Figure 6 and Table 3. The GC–MS analysis was performed to identify the major constituents of the prepared silver-loaded *T. turbinata* oil nanoemulsion (Ag-TTO-NE) and estimate their relative chromatographic abundances based on peak area percentages. The reported percentages therefore represent relative abundances rather than absolute quantitative concentrations.

The chromatographic profile showed a chemically varied makeup, dominated by lipid-derived chemicals. The detected contents were fatty acids, fatty acid esters, oxygenated chemicals, heterocyclic compounds, and minor sterol-related substances. This compositional heterogeneity is in agreement with prior observations on the biochemical complexity of brown algal lipid extracts, containing many lipid subclasses that influence the physicochemical features of the algal-derived formulations [47,48].

The most prevalent discovered component was dodecanoic acid, 4-penten−1-yl ester (19.43%), followed by isosorbide (8.43%), hexadecanoic acid (5.53%), dodecanoic acid (2.20%), oleic acid (1.61%), and various 9-octadecenoic acid derivatives. The abundance of fatty acids and their esters is in agreement with the lipid-rich nature of brown algae and with previous observations on the chemical composition of *Turbinaria* species [48].

Also found were several esterified lipid molecules, such as octanoic acid ester and derivatives of hexadecenoic acid ester and 9-octadecenoic acid, 1,2,3-propanetriyl ester. These are typically found in extracts of algal lipids and are usually related to the metabolism of glycerolipids and store lipids [48]. The existence of oxygenated low-molecular-weight metabolites was suggested by the detection of isosorbide (oxygen-containing heterocyclic molecule) at 8.43%. Also discovered was ethyl iso-allocholate (1.63–1.92%) and several minor sterol-related chemicals. Sterol-derived compounds are well-known components of brown algal lipids and contribute to the structural diversity of lipidomes of algae [36].

The GC–MS analysis showed a complex lipid-rich composition of the Ag-TTO-NE formulation, mostly constituted by fatty acids and their ester derivatives, coupled with oxygenated and sterol-related chemicals. These results provide qualitative information on the main components of the examined lipid fraction. Thus, the total evidence from GC–MS, FTIR, DLS, zeta potential, TEM, and EDX investigations was used to interpret the physicochemical characterization of the nanoemulsion, rather than only using GC–MS.

Species of genus *Turbinaria* have been previously found to possess a wide range of bioactive ingredients, including fatty acids, sterols, hydrocarbons, amino acids and other secondary metabolites [49].

### 2.3. Molecular Docking Analysis

#### 2.3.1. Ligand–Protein Interactions and Molecular Docking Analysis

To investigate the possible molecular mechanisms underlying the anticancer, antioxidant and antimicrobial activities of the *T. turbinata* oil nanoemulsion, the major compounds identified by GC–MS analysis were docked against three therapeutically relevant targets, namely the thioesterase domain of human fatty acid synthase (FASN; PDB ID: 7MHD), Kelch-like ECH-associated protein 1 (KEAP1; PDB ID: 4L7B), and DNA gyrase subunit B ATPase domain (GyrB; PDB ID: 3U2K). These proteins were selected because of their established roles in cancer progression, oxidative stress regulation, and bacterial survival, respectively [50,51,52].

**Figure 7:**
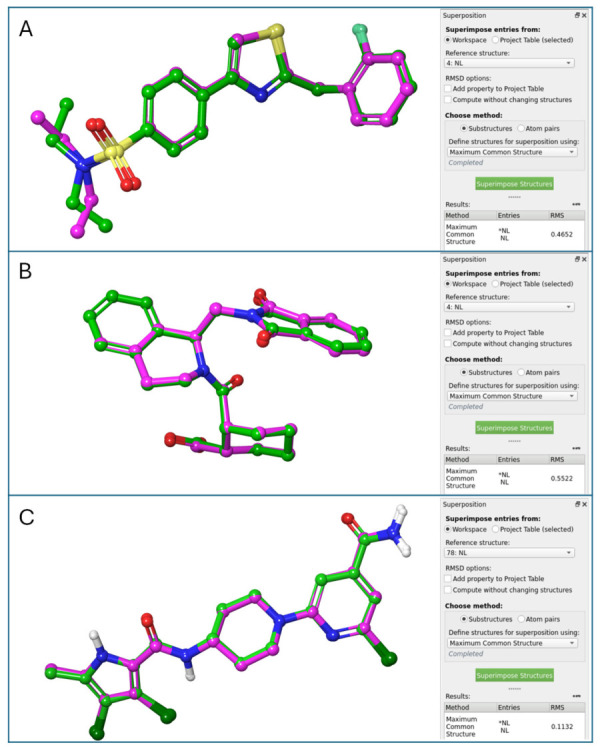
Three-dimensional superposition of the re-docked ligands (magenta) and the co-crystallized ligands (green) for (**A**) FASN, (**B**) KEAP1, and (**C**) GyrB. Atom colors are as follows: carbon, green (co-crystallized ligands) or magenta (re-docked ligands); nitrogen, blue; oxygen, red; sulfur, yellow; fluorine, dark green; and hydrogen, white.

Prior to docking, the docking protocol was validated by re-docking the co-crystallized ligands into their corresponding active sites. The obtained RMSD values were 0.4652 Å for FASN, 0.5522 Å for KEAP1, and 0.1132 Å for GyrB, all of which were substantially below the accepted threshold of 2.0 Å (Figure 7A–C). These results confirmed the reliability and reproducibility of the docking methodology and demonstrated that the generated docking poses closely reproduced the experimentally observed ligand conformations.

Molecular docking of the three-dimensional structures of the marine compounds was performed using the Extra Precision (XP) mode. For each protein target, multiple scoring functions, including Gscore, Emodel, XP Gscore, and MMGBSA ΔG Bind, were calculated to assess binding affinity and ligand ranking. The obtained docking scores for both the marine compounds and reference ligands are summarized in Table 4, Table 5 and Table 6.

#### 2.3.2. Interaction with Fatty Acid Synthase (FASN)

Among the investigated targets, FASN exhibited the highest affinity toward several compounds present in the nano-emulsion. The co-crystallized ligand showed a Glide XP score of −10.717 kcal/mol and an MM-GBSA binding energy of −118.95 kcal/mol. Notably, Octadecanoic acid, 4-hydroxy-, methyl ester emerged as the most promising phytochemical constituent, displaying a docking score of −9.084 kcal/mol and a favorable binding free energy (ΔG_bind) of −107.55 kcal/mol. Other compounds, including Dodecanoic acid, 4-penten−1-yl ester and octanoic acid, 1-methyltridecyl ester, also demonstrated considerable binding affinities, with docking scores of −7.377 and −7.845 kcal/mol, respectively (Table 4).

**Figure 8:**
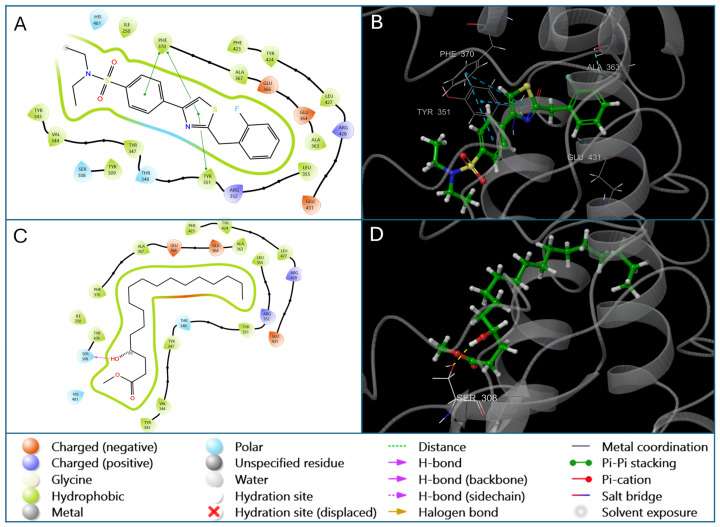
The 2D and 3D binding modes of the co-crystallized ligand (**A**,**B**) and octadecanoic acid, 4-hydroxy-, methyl ester (**C**,**D**) within the binding pocket of FASN (PDB: 7MHD). In the 3D representations, yellow dashed lines indicate hydrogen bonds, cyan dashed lines indicate aromatic hydrogen bonds, and blue dashed lines indicate pi–pi stacking interactions. The ligand is shown as green sticks, and the protein is displayed as a gray cartoon.

Analysis of the binding mode revealed that Octadecanoic acid, 4-hydroxy-, methyl ester occupied the catalytic cavity of FASN and established multiple hydrophobic interactions with residues lining the substrate-binding channel. Furthermore, the hydroxyl functional group contributed to stabilizing the ligand through hydrogen-bond interactions with Ser308 residues (Figure 8). These interactions may interfere with the enzymatic activity of FASN, a key enzyme involved in de novo fatty acid biosynthesis that is frequently overexpressed in numerous cancers. Inhibition of FASN has been associated with the suppression of tumor cell proliferation, induction of apoptosis, and reduction of metastatic potential, suggesting that the observed cytotoxic activity of the nano-emulsion may partly originate from interference with this metabolic pathway [53,54].

#### 2.3.3. Interaction with KEAP1

KEAP1 is a key negative regulator of the Nrf2 antioxidant signaling pathway and has emerged as an attractive therapeutic target in oxidative stress-related disorders and cancer. Docking analysis revealed that Octadecanoic acid, 4-hydroxy-, methyl ester exhibited the strongest predicted interaction among the identified compounds, with a docking score of −5.876 kcal/mol and an MM-GBSA binding energy of −78.13 kcal/mol. Isosorbide also demonstrated moderate affinity toward KEAP1, with a docking score of −5.476 kcal/mol (Table 5).

**Figure 9:**
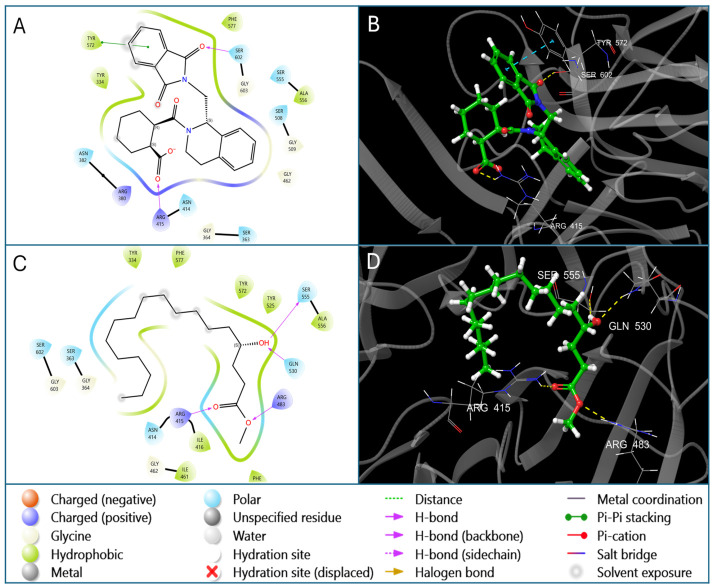
The 2D and 3D binding modes of the co-crystallized ligand (**A**,**B**) and octadecanoic acid, 4-hydroxy-, methyl ester (**C**,**D**) within the binding pocket of KEAP1 (PDB: 4L7B). In the 3D representations, yellow dashed lines indicate hydrogen bonds, and blue dashed lines indicate pi–pi stacking interactions. The ligand is shown as green sticks, and the protein is displayed as a gray cartoon.

Analysis of the binding poses indicated that Octadecanoic acid, 4-hydroxy-, methyl ester was accommodated within the KEAP1 binding pocket and established a network of hydrophobic and polar interactions. The hydroxyl group formed hydrogen bonds with Gln530 and Ser555, while the ester moiety interacted through hydrogen bonding with Arg415 and Arg483 (Figure 9). Although the binding affinity was lower than that observed for FASN, the interaction profile suggests the potential to modulate KEAP1–Nrf2 signaling. Activation of the Nrf2 pathway by disruption of KEAP1 interactions is known to enhance cellular antioxidant defenses and reduce oxidative damage. Therefore, the experimentally observed antioxidant activity of the nano-emulsion may be partially explained by interactions between its lipid constituents and the KEAP1 binding site [55].

#### 2.3.4. Interaction with DNA Gyrase B

To evaluate the potential antibacterial mechanism of the nano-emulsion, the major constituents were docked against the ATP-binding domain of bacterial DNA gyrase B, an essential enzyme involved in DNA replication and transcription. The native ligand exhibited a docking score of −4.936 kcal/mol, whereas 9-octadecenoic acid (oleic acid) showed the highest affinity among the phytochemicals, with a docking score of −4.700 kcal/mol and a binding free energy of −85.09 kcal/mol. Isosorbide and octanoic acid, 1-methyltridecyl ester followed closely with docking scores of −4.545 and −4.454 kcal/mol, respectively (Table 6).

**Figure 10:**
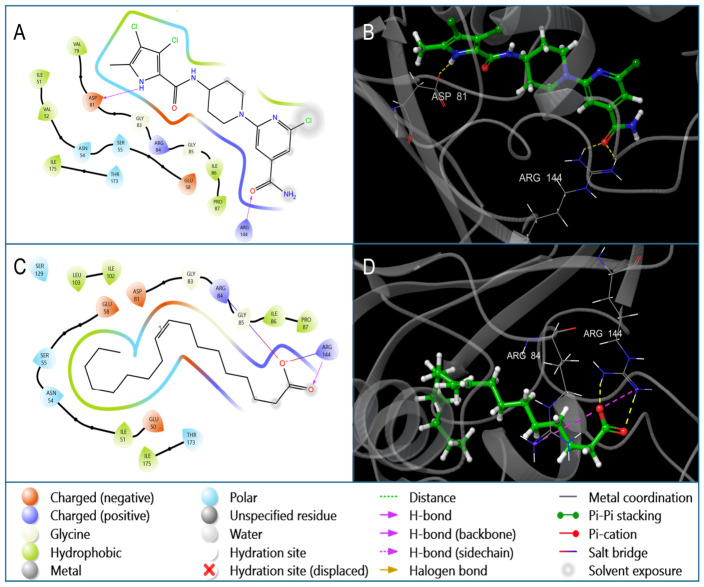
The 2D and 3D binding modes of the co-crystallized ligand (**A**,**B**) and 9-octadecenoic acid (Z) (oleic acid) (**C**,**D**) within the binding pocket of DNA gyrase (PDB: 3U2K). In the 3D representations, yellow dashed lines indicate hydrogen bonds and magenta dashed lines indicate salt bridges. The ligand is shown as green sticks, and the protein is displayed as a gray cartoon.

The docking pose of oleic acid revealed extensive hydrophobic interactions throughout the ATP-binding cavity, enabling its long aliphatic chain to occupy a substantial portion of the active-site groove. In addition, the carboxylate moiety formed a hydrogen bond with Arg144 and electrostatic interactions with positively charged residues within the binding pocket, contributing to the stability of the ligand–protein complex (Figure 10). Such interactions may hinder ATP binding and consequently impair DNA gyrase catalytic activity. Since DNA gyrase is absent in mammalian cells but essential for bacterial survival, inhibition of this enzyme represents an established antibacterial strategy [56]. The observed docking results are consistent with the strong antimicrobial activity demonstrated by the nano-emulsion against both Gram-positive and Gram-negative bacterial strains, including *Bacillus subtilis*, *Staphylococcus aureus*, *Pseudomonas aeruginosa*, and *Salmonella typhi*.

#### 2.3.5. Structure–Activity Relationship of the Major Bioactive Constituents

Comparison of the docking results across the three biological targets revealed that long-chain fatty acid derivatives generally exhibited superior binding affinities compared with smaller oxygenated compounds. In particular, Octadecanoic acid, 4-hydroxy-, methyl ester consistently ranked among the highest-scoring compounds against both FASN and KEAP1, while oleic acid demonstrated the strongest interaction with DNA gyrase B. The enhanced binding affinity of these molecules can be attributed to their long hydrophobic carbon chains, which maximize van der Waals interactions within the largely hydrophobic binding pockets, together with the presence of polar functional groups capable of forming hydrogen bonds.

Collectively, the molecular docking findings suggest that the biological activities of the *T. turbinata* oil nanoemulsion arise from a synergistic contribution of multiple lipid-derived constituents rather than a single active compound. The strong interactions observed with FASN support anticancer activity, whereas favorable binding toward DNA gyrase B provides a mechanistic explanation for the antibacterial effects. Moreover, modulation of KEAP1 may contribute to the antioxidant properties observed experimentally. These computational findings complement the in vitro biological assays and support the potential application of *T. turbinata* oil nanoemulsions as multifunctional nanotherapeutic agents.

### 2.4. Predicted Drug-likeness and ADMET Properties

The pharmacokinetic profiles of the major *T. turbinata* oil constituents were evaluated using the QikProp module (Table 7); the complete set of QikProp descriptors for all investigated compounds is provided in Supplementary Table S1. Most compounds exhibited low #Stars values, indicating that the majority of their physicochemical and pharmacokinetic descriptors fell within the recommended ranges for known drugs. Molecular weight (Mol_MW), hydrogen-bond donor (DonorHB), hydrogen-bond acceptor (AccptHB), solvent-accessible surface area (SASA), and reactive functional group counts (#RtvFG) were generally consistent with accepted drug-likeness criteria. In addition, the predicted lipophilicity (QplogPo/w) and aqueous solubility (QplogS) values suggested favorable membrane permeability, although some long-chain fatty acid derivatives exhibited reduced aqueous solubility due to their hydrophobic nature.

Furthermore, the evaluated compounds demonstrated acceptable safety and absorption characteristics, with most QPlogHERG values indicating a low risk of cardiotoxicity. Predicted blood–brain barrier permeability (QplogBB) and central nervous system (CNS) activity ranged from low to moderate, while QplogKhsa and #Metab values suggested favorable plasma protein binding and metabolic stability. Importantly, the majority of compounds showed moderate to high predicted human oral absorption, supporting their potential as orally bioavailable bioactive agents. Collectively, these findings indicate that the principal constituents of *T. turbinata* oil possess favorable predicted drug-like and pharmacokinetic properties, supporting their further investigation as potential antimicrobial and anticancer agents. However, these computational predictions apply exclusively to the individual compounds and should not be extrapolated to the Ag-TTO-NE nanoemulsion as an integrated formulation, whose pharmacokinetic behavior and biological performance are influenced by both its chemical composition and formulation-specific physicochemical characteristics.

### 2.5. Anticancer Activity

**Figure 11:**
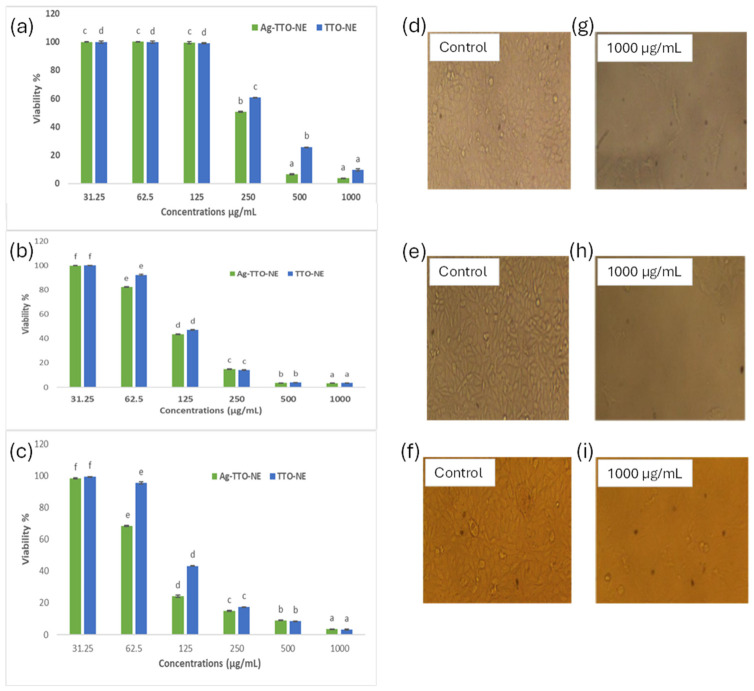
Effect of Ag-TTO-NE and TTO-NE on cell viability and morphology. Cell viability (%) of (**a**) normal human fibroblast (Wi38), (**b**) human breast cancer (MCF−7), and (**c**) human ovarian cancer (SKOV−3) cells following treatment with increasing concentrations of Ag-TTO-NE and TTO-NE. Representative phase-contrast micrographs showing untreated control cells (**d**–**f**) and cells treated with 1000 μg/mL Ag-TTO-NE are presented for Wi38, MCF−7, and SKOV−3 cell lines (**g**–**i**), respectively. Different letters indicate statistically significant differences among concentrations within the same nanoemulsion formulation (*p* < 0.05).

Figure 11 shows the effect of the silver-loaded *T. turbinata* oil nanoemulsion (Ag-TTO-NE) and *T. turbinata* oil nanoemulsion (TTO-NE) on the viability percentage of the normal human fibroblast cell line (Wi38), human breast cancer cell line (Mcf7), and ovarian cancer cell line (SKOV3) cells. The results demonstrate that increased concentrations of the Ag-algal oil nanoemulsion (Ag-TTO-NE) and algal oil nanoemulsion (TTO-NE) increased the cytotoxic response against normal and cancerous cell lines. The progressive decline in cellular viability was observed with increasing nanoemulsion concentration, confirming the dose-dependent biological activity of the nanoemulsion and silver-algal oil-loaded nanoemulsion.

The greater reduction in the cell viability of normal (Wi38), human breast cancer cell line (Mcf7), and ovarian cancer cell line (SKOV3) cells, observed at 500 and 1000 µg/mL of (Ag-TTO-NE), suggests that silver incorporation enhanced the antiproliferative activity of the nanoemulsion under the experimental conditions. Ag-TTO-NE demonstrated enhanced cytotoxic activity compared with TTO-NE, particularly in MCF−7 and SKOV3 cells at intermediate concentrations, suggesting that silver incorporation improved the antiproliferative efficacy of the nanoemulsion.

The results of Figure 11d–i show the microscopic examination of normal and cancer cell line cells after being treated with Ag-TTO-NE and the control. The results demonstrate the untreated control cells retained normal morphology and confluency, whereas the treated cells showed reduced cell density, altered cellular architecture, shrinkage, and increased detachment, particularly at higher concentrations. These morphological changes were consistent with concentration-associated cytotoxic stress.

**Figure 12:**
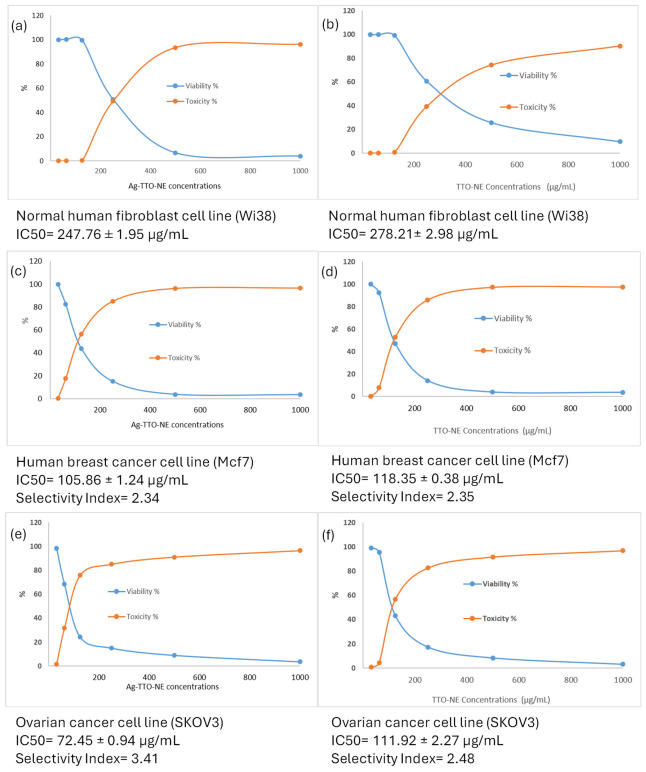
Cell viability (%), cytotoxicity (%), IC_50_ values, and selectivity indices of Ag-TTO-NE and TTO-NE against (**a**,**b**) normal human fibroblast (Wi38), (**c**,**d**) human breast cancer (MCF−7), and (**e**,**f**) human ovarian cancer (SKOV−3) cell lines. Panels (**a**,**c**,**e**) represent Ag-TTO-NE treatment, whereas panels (**b**,**d**,**f**) represent TTO-NE treatment.

Figure 12 shows the cytotoxic activity of Ag-TTO-NE and TTO-NE as tested against normal human fibroblasts (Wi38), human breast cancer cells (MCF−7) and human ovarian adenocarcinoma cells (SKOV3). Both formulations displayed more toxicity against cancer cells compared to normal fibroblasts, thereby disclosing a preferential cytotoxic activity under the experimental conditions.

The IC50 values of Ag-TTO-NE and TTO-NE on Wi38 cells were 247.76 ± 1.95 and 278.21 ± 2.98 µg/mL, respectively, indicating relatively lower cytotoxicity toward normal cells than toward the cancer cell lines. In the cancer cell lines, much lower IC50 values were obtained. Ag-TTO-NE and TTO-NE exhibited IC50 values of 105.86 ± 1.24 and 118.35 ± 0.38 µg/mL on MCF−7 cells, respectively. Ag-TTO-NE showed lower IC50 values (72.45 ± 0.94 µg/mL) than TTO-NE (111.92 ± 2.27 µg/mL) on SKOV3 cells, with both formulations showing significant activity.

Further selectivity indices also showed preferential toxicity to cancer cells. The selectivity indices of Ag-TTO-NE against MCF−7 and SKOV3 cells were 2.34 and 3.41, respectively. In contrast, the selectivity indices of TTO-NE against MCF−7 and SKOV3 cells were 2.35 and 2.48, respectively. Among the tested formulations, Ag-TTO-NE exhibited the highest selectivity for SKOV3 cells (SI = 3.41), indicating an improved activity against ovarian cancer cells upon the addition of silver. Nevertheless, these SI values should be regarded as preliminary indicators of selectivity, and further biological investigations are required to confirm their therapeutic significance.

The observed cytotoxicity profile suggests that both nanoemulsion formulations preferentially target cancer cells, with reduced toxicity towards the normal fibroblasts. The much higher IC50 values found for Wi38 cells compared to MCF−7 and SKOV3 cells indicate lower cytotoxicity toward normal cells and preferential cytotoxicity under the experimental conditions. The effect of silver inclusion was different depending on the type of cancer cell. The IC50 values of both formulations in MCF−7 cells were very similar, showing that silver nanoparticles did not significantly improve the cytotoxic action against breast cancer cells in the current experimental settings. This result indicates that the bioactive ingredients of the algal oil nanoemulsion may contribute substantially to the antiproliferative action in MCF−7 cells. Ag-TTO-NE showed better activity against SKOV3 cells, with lower IC50 and a higher selectivity index than the nanoemulsion without silver. The silver incorporation was associated with enhanced activity against SKOV3 but had minimal effect against MCF.

This increased reaction may suggest the increased sensitivity of ovarian cancer cells to the combination effect of silver nanoparticles and algal lipid-derived metabolites. Previous research has indicated that silver nanoparticles can improve the efficiency of bioactive natural products by boosting reactive oxygen species production, mitochondrial dysfunction, membrane damage and apoptotic signaling.

AgNPs synthesized by *Sargassum subrepandum brown alga* exhibited significant antimicrobial activity against *Pseudomonas aeruginosa*, showing cytotoxic effects on MCF−7 breast adenocarcinoma cells. The results denoted a decline in cell viability and showed morphological changes in the cancer cells [56].

### 2.6. Antioxidant Activity

**Figure 13:**
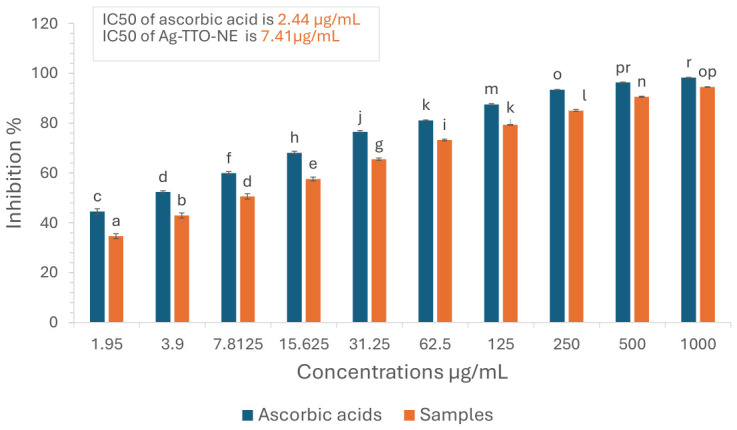
Antioxidant activity of silver-loaded *T. turbinata* oil nanoemulsion in comparison to ascorbic acid as a positive control. Bars represent standard error (SE), with different letters are significantly different from each other (*p* < 0.05).

Figure 13 presents the antioxidant activity of the Ag-algal nanoemulsion (Ag-TTO-NE) in comparison to ascorbic acid as a reference. The results showed that the antioxidant activity of Ag-TTO-NE increased with the increase of its concentration, but it was still lower than the antioxidant activity of ascorbic acid. The antioxidant activity of Ag-TTO-NE is 94.55% at the concentration of 1000 µg/L, with ascorbic acid at 98.29%. In addition, the results demonstrate that the IC50 of Ag-TTO-NE is 7.41 µg/mL compared to ascorbic acid at 2.44 µg/mL, indicating that Ag-TTO-NE has high antioxidant activity. *Turbinaria* brown alga is rich in phytochemical and pharmacological diversity and possesses anticancer, cardioprotective, and antioxidant properties [48].

### 2.7. Antibacterial Activities

**Figure 14:**
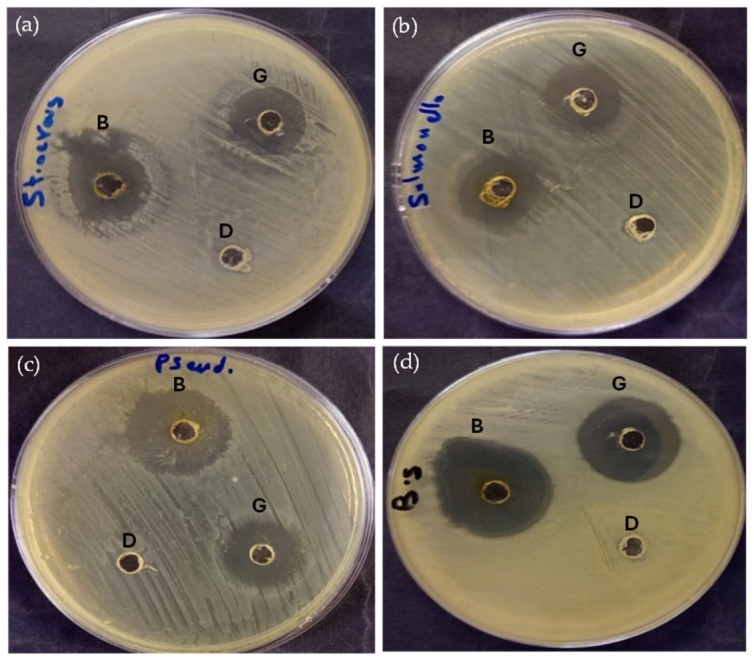
Antibacterial activity of silver-loaded *T. turbinata* oil nanoemulsion against (**a**) *Staph. aureus* (*ATCC 6538*), (**b**) *Salmonella typhi* (*ATCC 6539*), (**c**) *Pseudomonas aeruginosa* (*ATCC90274*), (**d**) *Bacillus subtilis* (*ATCC 6633*). B, bacteria; G, Gentamicin (as positive control); D, dimethyl sulfoxide (DMSO) negative control.

The results in Figure 14 and Table 8 show the antibacterial activity of the silver-loaded *T. turbinata* oil nanoemulsion (Ag-TTO-NE) and Gentamicin antibiotic as a reference against *B. subtilis* (ATCC 6633) and *S. aureus* (ATCC 6538) Gram-positive bacteria, and *P. aeruginosa* (*ATCC90274*) and *Salmonella typhi* (ATCC 6539) Gram-negative bacteria, as measured by the clear zone (mm).

The results indicate that the Ag-algal nanoemulsion (Ag-TTO-NE) exhibited significant antibacterial activity against *B.subtilis* (ATCC 6633) and *S. aureus* (ATCC 6538) Gram-positive bacteria, and *P. aeruginosa* (*ATCC90274*) and *S. typhi* (ATCC 6539) Gram-negative bacteria. In the agar diffusion assay, clear zones ranged from 18–28 mm, with the strongest activity against *B. subtilis* (28 ± 1.0 mm) followed by *Pseudomonas aeruginosa* (25 ± 0.7 mm), *S. aureus* (20 ± 0.5 mm), and *Salmonella typhi* (18 ± 0.4 mm) compared to the antibiotic control, which possessed (25 ± 0.6; 19 ± 0.8; 18 ± 0.2; and 17 ± 0.1), respectively. The Ag-algal nanoemulsion has high antibacterial activity against *P. aeruginosa* (*ATCC90274*), with a clear zone of (25 ± 0.7 mm) compared to the antibiotic control (18 ± 0.2 mm).

The results in Table 9 exhibit MIC, MBC and the MBC/MIC Index of the Ag-algal oil nanoemulsion (Ag-TTO-NE) against *B. subtilis* (ATCC 6633) and *S. aureus* (ATCC 6538) Gram-positive bacteria, and *P. aeruginosa* (*ATCC90274*) and *Salmonella typhi* (ATCC 6539) Gram-negative bacteria. MIC analysis showed inhibitory concentrations of 15.62 µg/mL for all tested bacteria except *Salmonella typhi* (*ATCC 6539*)*,* which possessed 31.25 µg/mL. The MBC value was 31.25 µg/mL for all tested bacteria, except *Salmonella typhi* (*ATCC 6539*)*,* (62.5 µg/mL). The consistent MBC/MIC ratio of two confirmed a bactericidal mode of action; these findings indicate that the Ag-algal oil nanoemulsion (Ag-TTO-NE) possesses effective broad-spectrum antibacterial activity and enhanced microbial killing potential. AgNPs synthesized by *Sargassum subrepandum* brown alga exhibited significant antimicrobial activity against *Pseudomonas aeruginosa* [56]. Brown algae are a naturally rich source of phenolic compounds, phlorotannins, carotenoids, sulfated polysaccharides and polyunsaturated fatty acids, which are known to have a major contribution to biological protection against oxidative and microbiological stresses [57]. The inclusion of these chemicals in nanoemulsion systems could boost dispersion stability, increase surface area and improve cellular penetration, hence improving their biological performance [58]. Silver nanoparticles derived from brown algae were reported to exhibit antioxidant, anticancer, and antibacterial activities against Gram-positive bacteria [23].

Furthermore, the hybrid nature of the system suggests a synergistic antimicrobial mechanism, where the algal bioactive compounds and silver nanoparticles act cooperatively. While AgNPs exert strong bactericidal effects through membrane disruption, protein denaturation, and reactive oxygen species (ROS) generation, algal metabolites may contribute additional antimicrobial pressure through metabolic interference and membrane permeability modulation. This synergism has been increasingly reported in green nanocomposite systems, where biological matrices enhance the efficacy and reduce the required dosage of metallic nanoparticles, thereby improving biosafety profiles [59]. The use of silver nanoparticles and oregano oil together in the form of nanoemulsions showed a synergistic impact and increased antibacterial and antifungal activities [60].

The present results strongly show that lipids derived from brown algae coated with silver nanoparticles (AgNPs) exhibited promising multifunctional bioactivity, with anticancer, antioxidant and antibacterial properties. This biological activity may be attributable to the synergistic effect of the intrinsic bioactive metabolites of brown algae and the specific physicochemical features of silver nanoparticles. Brown algae are naturally rich in phlorotannins, fucoidan, carotenoids, polyunsaturated fatty acids, and other phenolic compounds with antioxidant and therapeutic potential. These metabolites also serve as lowering and stabilizing agents in green synthesis, which enhances the stability and biological efficiency of nanoparticles [61]. The cytotoxic activity that was observed against the human breast cancer cell line (Mcf7) and ovarian cancer cell line (SKOV3) cells may indicate that the nanoemulsion can exert antitumor effects against cancers with different molecular and biological characteristics. The boosted activity may be endorsed to improve the intracellular delivery of bioactive compounds mediated by the nanoemulsion system, in addition to the cytotoxic properties of silver nanoparticles [10].

### 2.8. Limitations of the Study

There are some limitations to the current study that need to be mentioned. Firstly, GC–MS analysis was performed to identify the main ingredients of the produced nanoemulsion and to estimate their relative chromatographic abundances, and hence it is not a representation of absolute quantitative chemical standardization. Second, the molecular docking and ADMET analyses were presented as exploratory computational tools to supplement the experimental findings and should not be taken as direct experimental validation of the postulated biological pathways. Finally, although the developed nanoemulsion showed promising physicochemical properties and in vitro biological activities, further quantitative chemical characterization, mechanistic studies, batch-to-batch reproducibility studies, and in vivo pharmacological validation are needed before therapeutic applications can be considered.

## 3. Materials and Methods

### 3.1. Alga

The marine alga *T. turbinata* was gathered from the Jeddah coast of the Red sea, Saudi Arabia, in May 2024 and then identified according to (Taylor, 1985). The corresponding author identified the species based on morphological characters using the taxonomic keys supplied by Taylor [62]. It was washed with tap water, then with DD water, and dried in the shade, subsequently ground, and dried in an electrical oven at 60 °C till consistent weight.

#### 3.1.1. Algal Extract

The lipid was extracted from 100 g of the finely ground particles of *T. turbinata* marine alga with 1 L of Hexane (≥99%, analytical grade, Merck, Darmstadt, Germany). The mixture was continuously stirred using a magnetic stirrer at 500 rpm for 2 h at room temperature. The mixture was filtered after extraction to separate the solid residues and the solvent phase. The obtained filtrate was then concentrated and dried under reduced pressure with a rotary evaporator for the solvent removal to obtain the crude lipid extract [26].

#### 3.1.2. Nanoemulsion Synthesis

Tween 80, Span 80, and silver nitrate (AgNO_3_) were purchased from Sigma-Aldrich (St. Louis, MO, USA). A mixture of surfactants (Span 80 and Tween 80 in the ratio 28:72 *v*/*v*) was prepared and well homogenized with magnetic stirring for uniform dispersion. Approximately one g of *T. turbinata* marine algal oil, extracted as shown in the previous stage, was taken, and about 4 mL of the surfactant mixture was added and stirred thoroughly to form a stable oil phase. For synthesis of the *T. turbinata* oil nanoemulsion (TTO-NE), 5 mL of water was added drop by drop, with continuously stirring using a magnetic stirrer (500 r/s) at room temperature. For synthesis of the silver-*T. turbinata* oil nanoemulsion (Ag-TTO-NE), 5 mL of silver nitrate (AgNO_3_) solution (0.017 mg/mL) was added instead of water [63].

The reaction mixture was stirred till a definite brown color was developed, showing the formation of silver nanoparticles due to the reduction of silver ions by the bioactive components of the algal oil.

### 3.2. Nanoparticle Characterization

#### 3.2.1. Fourier Transform Infrared (FTIR) Spectroscopy

The silver-loaded *T. turbinata* oil nanoemulsion (Ag-TTO-NE), *T. turbinata* oil nanoemulsion (TTO-NE), and hexane extract were characterized by Fourier-transform infrared (FTIR) spectroscopy in the range 400–4000 cm^−1^ using an FT-IR 5300 spectrophotometer (JASCO Europe S.r.l., Cremella, Italy). Before analysis, the samples were dried to remove residual moisture and solvent traces.

#### 3.2.2. Zeta Potential and Zetasizer Analysis

The particle size, PDI, and zeta potential of Ag-TTO-NE were assessed to evaluate the physicochemical characteristics, colloidal stability, and homogeneity. The average hydrodynamic diameter and size uniformity of the nanoemulsion droplets were determined by the particle size distribution and PDI, while the surface charge and stability of the dispersed system were analyzed by the zeta potential. All measurements were performed by dynamic light scattering (DLS) with a Zetasizer Nano ZS90 (Malvern Instruments Ltd., Malvern, UK) under standard operating conditions.

#### 3.2.3. Energy-Dispersive Spectroscopy (EDS)

The surface morphology and elemental contents of Ag-TTO-NE were investigated using field emission scanning electron microscopy (SEM) coupled with energy-dispersive spectroscopy (JEOL JSM−6510/v, Tokyo, Japan).

#### 3.2.4. Transmission Electron Microscopy (TEM)

The particle sizes and shapes of Ag-TTO-NE were determined using TEM (JEOL JSM−6510/v, Tokyo, Japan).

### 3.3. GC–MS Analysis

The lipid extract of Ag-TTO-NE (silver-loaded *T. turbinata* oil nanoemulsion) was characterized by transesterification using 0.5 N methanolic KOH. Briefly, 0.02 g of each lipid extract was mixed with methanolic KOH and heated at 50 °C for 15 min. The reaction mixture was cooled, acidified with 0.4 N HCl, and extracted with petroleum ether/hexane (1:1, *v*/*v*). The upper organic layer containing the fatty acid methyl esters (FAMEs) was recovered, dried at 40 °C, reconstituted in hexane, and injected into the GC–MS apparatus [26].

The chemical composition of the Ag-TTO-NE extract was analyzed by gas chromatography–mass spectrometry (GC–MS) using an ISQ™ 7000 system (Thermo Fisher Scientific, Waltham, MA, USA) operating in electron ionization (EI) mode at 70 eV. A 1 µL aliquot of the extract was injected in split mode (split flow: 20 mL/min). Helium (99.999% purity) was used as the carrier gas at a constant flow rate of 1.5 mL/min. The injector, transfer line, and ion source temperatures were maintained at 250, 240, and 250 °C, respectively. Mass spectra were recorded over an *m*/*z* range of 40–900.

The oven temperature program was as follows: the initial temperature of 50 °C was held for 2 min, then increased to 200 °C at 4 °C/min and held for 2 min, followed by an increase to 300 °C at 5 °C/min and held for 5 min. Compounds were tentatively identified by comparing their mass spectra with those in the NIST and Wiley mass spectral libraries.

### 3.4. Docking Study

#### 3.4.1. Protein Preparation

The three-dimensional crystal structures of the selected target proteins were retrieved from the Protein Data Bank (PDB) database. The investigated proteins included the thioesterase domain of human fatty acid synthase (FASN-TE, PDB ID: 7MHD), Kelch-like ECH-associated protein 1 (KEAP1, PDB ID: 4L7B), and the ATPase domain of DNA gyrase subunit B (GyrB, PDB ID: 3U2K). Protein structures were prepared using the Protein Preparation Wizard implemented in Schrödinger Maestro (Schrödinger LLC, New York, NY, USA). During preparation, bond orders were assigned, hydrogen atoms were added to all residues, missing side chains and loops were corrected where necessary, and protonation states were optimized under physiological conditions (pH 7.0 ± 2.0). Water molecules located more than 5 Å away from the ligand-binding region were removed, while those involved in ligand recognition were retained. Finally, restrained energy minimization was performed using the OPLS4 force field until convergence was achieved, to remove steric clashes and optimize the geometry of the protein structures.

#### 3.4.2. Ligand Preparation

The major compounds identified in the GC–MS profile of *T. turbinata* oil were selected for molecular docking analysis. Ligand structures were generated and prepared using the LigPrep module of Schrödinger. The preparation protocol included the conversion of two-dimensional structures into energetically minimized three-dimensional conformations, assignment of bond orders, generation of possible ionization states, stereoisomers, and tautomeric forms at physiological pH (7.0 ± 2.0) using Epik. Geometry optimization was subsequently performed using the OPLS4 force field to ensure biologically relevant ligand conformations prior to docking.

#### 3.4.3. Grid Generation and Molecular Docking

The active sites of the selected target proteins were identified based on the coordinates of their respective co-crystallized ligands. Receptor grids were generated using the Receptor Grid Generation module in Schrödinger Maestro, with each grid centered on the native ligand within the binding pocket. Prior to docking, the reliability of the docking protocol was validated by re-docking the co-crystallized ligands into their corresponding active sites. Subsequently, molecular docking of the compounds, including the constituents of *T. turbinata* oil, was performed using the Glide Ligand Docking module in Extra Precision (XP) mode, while all other parameters were maintained at their default settings to ensure accurate prediction of ligand–protein interactions.

#### 3.4.4. Binding Free Energy Estimation

To further evaluate ligand-binding affinity, Molecular Mechanics-Generalized Born Surface Area (MM-GBSA) calculations were performed using the Prime MM-GBSA module implemented in Schrödinger. The binding free energy (ΔG_bind) was calculated according to

ΔG_bind = G_complex − (G_protein + G_ligand)

where G_complex, G_protein, and G_ligand represent the minimized free energies of the protein–ligand complex, isolated protein, and isolated ligand, respectively. More negative ΔG_bind values indicate stronger and more favorable binding interactions.

### 3.5. In Silico ADMET Profiling

The pharmacokinetic and drug-likeness properties of the major *T. turbinata* oil constituents were predicted using the QikProp module of Schrödinger, which provides quantitative estimates of physicochemical, pharmacokinetic, and toxicity-related descriptors relevant to early-stage drug discovery. ADMET analysis was performed on the major compounds identified by GC–MS following individual structure preparation. Since currently available in silico ADMET tools are designed to evaluate discrete chemical entities rather than complex nanoformulations, the calculations were performed exclusively to assess the intrinsic drug-like and pharmacokinetic characteristics of the individual constituents.

### 3.6. Cytotoxicity on Cells

#### 3.6.1. MTT Assay

Cell-based experiments were performed by Science Way for Scientific Research and Consultations, Giza, Egypt. The human breast adenocarcinoma cell line (MCF-7), human ovarian adenocarcinoma cell line (SKOV3), and normal human fibroblast cell line (Wi38) were obtained from VACSERA (The Holding Company for Biological Products and Vaccines, Giza, Egypt).

The cytotoxic activity of the sample under study was assessed by the MTT colorimetric assay, with minor modifications, based on prior procedures [60]. Cells were planted in sterile 96-well tissue culture plates with a density of 1 × 10^5^ cells/mL, and 100 µL of cell suspension was added to each well and incubated at 37 °C for 24 h for cell attachment and monolayer formation. The growth solution was aspirated after incubation, and the cell monolayers were cleaned twice with sterile washing medium.

For cytotoxicity evaluation, each Ag-TTO-NE or TTO-NE stock formulation was serially diluted in culture medium to obtain final concentrations ranging from 31.25 to 1000 µg/mL. Concentrations are expressed as the total mass concentration of the nanoemulsion formulation Ag-TTO-NE or TTO-NE.

Serial two-fold dilutions of the tested sample were made in RPMI−1640 maintenance medium containing 2% fetal bovine serum (FBS). Thereafter, 100 µL of each dilution was transferred into the respective wells, and control wells without any treatment were filled with only maintenance medium. Plates were incubated under conventional culture conditions (37 °C, 5% CO_2_) and evaluated microscopically for evident cytotoxic manifestations, including cellular rounding, shrinkage, granulation, detachment, and monolayer disruption.

The MTT solution (5 mg/mL in phosphate-buffered saline, PBS; Bio Basic Canada Inc., Markham, ON, Canada) was newly made to determine the viability. Then, 20 µL of MTT reagent was added to each well and shaken at 150 rpm for 5 min and then incubated at 37 °C in 5% CO_2_ for 4 h. After incubation, the supernatant was removed, and the formazan crystals formed were dissolved in 200 µL dimethyl sulfoxide (DMSO). Absorbance was determined at 560 nm, with background adjustment at 620 nm. Optical density readings were used directly as a measure of cell viability.

#### 3.6.2. Morphological Assessment of Cytotoxicity

Morphological changes related with cytotoxicity were examined using inverted light microscopy and established criteria for cell death [64]. Cellular structural changes were assessed, including cell volume decrease, cell membrane breakdown, cytoplasmic granulation, loss of adhesion, and nuclear modifications. Necrotic cells were defined by nuclear enlargement and disorganization of chromatin. Apoptotic cells were characterized by cell shrinkage, chromatin condensation, and nuclear fragmentation.

### 3.7. Antioxidant Activity (DPPH)

The antioxidant activity of Ag-TTO-NE was studied by 2,2-diphenyl−1-picrylhydrazyl (DPPH) (Sigma-Aldrich, St. Louis, MO, USA), radical scavenging assay as reported previously by Rinaldi et al. [64], with modifications.

Briefly, 0.004% (*w*/*v*) of methanolic solution of DPPH was freshly prepared. One mL aliquots of various concentrations of the tested material (1.9–1000 µg/mL) were added to 4 mL of DPPH solution. The reaction mixtures were incubated in the dark at room temperature for 30 min to permit interaction of antioxidant chemicals and DPPH radicals. After incubation, the absorbance of each sample was measured at 517 nm with a UV–visible spectrophotometer using a methanol blank. The radical scavenging activity was determined by using the following formula:

% Inhibition = [(Abs_control − Abs_sample)/Abs_control] × 100

where Abs_control is the absorbance of the DPPH solution without the sample, and Abs_sample is the absorbance in the presence of the tested sample.

### 3.8. Antibacterial Activity (MIC and MBC)

Antimicrobial activity of Ag-TTO-NE was investigated against Gram-positive bacteria, *Bacillus subtilis* (ATCC 6633) and *Staphylococcus aureus* (ATCC 6538), and Gram-negative bacteria, *Pseudomonas aeruginosa* (ATCC 90274) and *Salmonella typhi* (ATCC 6539). The antibacterial activity was mostly determined by the agar well diffusion technique. Briefly, Mueller–Hinton agar plates (150 mm) were inoculated with ~50 µL of bacterial suspension (10^6^ CFU/mL) evenly spread over the surface of the agar. Wells (4 mm diameter) were then prepared aseptically, and 50 µL of Ag-TTO-NE was put into the well. Standard antibiotics were employed as the positive control (Gentamicin(, and DMSO as the negative control. The plates were incubated at 30 °C for 24 h, and zones of inhibition were evaluated for antibacterial activity.

The antibacterial activity of Ag-TTO-NE was further tested by the broth microdilution method according to CLSI M07 and ISO 20776−1, with minor modifications. The test sample was serially diluted two-fold (1000–1.95 µg/mL) in Tryptic Soy Broth (TSB) and was added to sterile 96-well microtiter plates (100 µL/well). Fresh bacterial colonies (18–24 h) were suspended in sterile saline or broth, adjusted to a 0.5 McFarland standard, and diluted to a final inoculum of approximately 5 × 10^5^ CFU/mL per well. Preparation was completed within 15 min of the inoculation. Wells containing bacterial inoculum but no test material served as positive growth controls, and wells containing broth and test material but no bacterial inoculation served as sterility controls. Plates were incubated aerobically at 35 ± 2 °C for 16 to 20 h. Bacterial growth was monitored visually and by measuring the absorbance at 630 nm with a microplate reader. The minimum inhibitory concentration (MIC) was determined as the lowest concentration at which no observable bacterial growth occurred. To determine the minimum bactericidal concentration (MBC), aliquots from the wells at and above the MIC were subcultured onto agar plates and calculated as the lowest concentration showing no visible bacterial growth.

### 3.9. Statistical Analysis

Analysis of the data was performed with proper statistical procedures to examine the differences between the concentrations of nanoparticles. Results are expressed as mean ± standard error (SE). The significant differences between concentrations were evaluated by one-way analysis of variance (one-way ANOVA). When significant differences were found, post hoc multiple comparison tests were used to determine pairwise differences. Statistical significance was defined as *p* < 0.05. All analyses were performed using IPM SPSS 27.

## 4. Conclusions

A natural emulsification method based on surfactant stabilization was used to prepare a silver-loaded nanoemulsion based on *T. turbinata* oil (Ag-TTO-NE). The Ag-TTO-NE displayed physicochemical properties such as nanoscale particle size (5–12 nm), low polydispersity index PDI (0.093), and moderate negative surface charge (−19.3 mV), suggesting favorable colloidal properties under the tested conditions. EDX analysis confirmed the presence of silver in the synthesized nanoparticles. The biological studies demonstrated activity against MCF−7 and SK-OV−3 cancer cell lines, antibacterial effects against both Gram-positive and Gram-negative bacteria, and antioxidant properties. These biological effects may be attributed, at least in part, to the combined contribution of the phytochemical constituents of *T. turbinata* oil and the incorporated silver nanoparticles. Molecular docking analysis further revealed favorable binding interactions between several major GC–MS-identified compounds and the selected therapeutic targets, including human fatty acid synthase (FASN), Kelch-like ECH-associated protein 1 (KEAP1), and DNA gyrase B (GyrB), supporting the experimental observations and suggesting possible molecular interactions that may contribute to the observed biological activities. Moreover, in silico ADMET analysis of the major GC–MS-identified compounds indicated generally favorable predicted drug-likeness and oral absorption profiles. These predictions apply only to the individual constituents and provide complementary information regarding their intrinsic pharmacokinetic potential; however, they should not be interpreted as representing the pharmacokinetic behavior of the Ag-TTO-NE nanoemulsion. The results indicate that *T. turbinata* silver nanoemulsions represent a promising platform for the further development of multifunctional bioactive nanocarriers. In conclusion, the present findings provide preliminary evidence of the biological potential of *T. turbinata*-based silver nanoemulsions as multifunctional bioactive nanoplatforms. However, their efficacy, safety, and therapeutic potential has to be confirmed by additional mechanistic, pharmacological, and in vivo research.

## Figures and Tables

**Table 1:** FTIR spectra analysis of Ag-TTO-NE, showing functional group-related wavenumbers.

	Wavenumbers (cm^−1^)				
Ag-TTO-NE	TTO-NE	Hexane	*Δν	Proposed Assignment	Functional Groups
3356		3351	+4	OH	Hydroxyl
2919		ND	-	C–H stretching	Aliphatic hydrocarbons
1632		1606	+26	C=C stretching	Unsaturated fatty acids
1451		1395	+56	Bending vibration of CH_2_	Aliphatic hydrocarbon associated with lipids
1356		ND	-	CH_3_ symmetric bending	Aliphatic hydrocarbon chains
1261		1264	−3	C–O stretching	Alcohol/ether
1076		ND		C–O–C stretching	Ether/polysaccharide-like groups
1048		1015	+33	C–O stretching	Alcohol/ether

ND: not detected; *Δν = (Ag-TTO-NE wavenumber − hexane extract wavenumber).

**Table 2:** Energy dispersive X-ray spectroscopy analysis silver-loaded *T. turbinata* oil nanoemulsion (Ag-TTO-NE).

Elements	C	O	Na	Mg	Ag
Weight %	8.9	63.43	21.86	5.67	0.12

**Table 3:** GC–MS analysis of Ag-loaded *T. turbinata* oil nanoemulsion (Ag-TTO-NE).

	RT	Compounds	Formula	%	Types
1	21.18	Isosorbide	C_6_H_10_O_4_	8.43	Organoheterocyclic
2	30.03	Dodecanoic acid	C_12_H_24_O_2_	2.20	Saturated fatty acid
3	41.08	Octanoic acid, oct−3-en−2-yl ester	C_16_H_30_O_2_	2.24	Saturated octanoyl chain esterified
4	42.98	Octanoic acid, 1-methyltridecyl ester	C_22_H_44_O_2_	1.34	Fatty alcohol esters
5	46. 47	2-Hexenoic Acid, 5-Hydroxy−3,4,4-trimethyl	C_8_H_16_O	2.06	Fatty acid derivative
6	47.98	2,2,3,3,4,4 Hexadeutero Octadecanal	C_18_H_30_D_6_O	1.32	Deuterated fatty aldehydes
7	50.75	Dodecanoic acid, 4-penten−1-yl ester	C_17_H_32_O_2_	19.43	Oxygenated hydrocarbons
8	50.94	Hexadecanoic acid, 1,5-pentanediyl ester	C_37_H_72_O_4_	1.89	Polyol fatty acid esters
9	51.88	2,2,3,3,4,4 Hexadeutero Octadecana	C_18_H_30_D_6_O	12.21	Deuterated fatty aldehyde
10	54.35	Hexadecanoic Acid	C_19_H_38_O_4_	5.53	Saturated fatty acid
11	55. 75	9-Octadecenoic acid (Z)/Oleic Acid	C_18_H_34_O_2_	1.61	Monounsaturated fatty acid
12	57.54	Octadecanoic acid, 4-hydroxy-, methyl ester	C_19_H_38_O_3_	2.61	Fatty acid methyl esters
13	58. 39	9-Octadecenoic Acid (Z)	C_18_H_34_O_2_	2.10	Monounsaturated omega-9 fatty acid
14	60.13	9-Octadecenoic Acid (Z)	C_18_H_34_O_2_	1.47	Monounsaturated omega-9 fatty acid
15	60.91	9-Octadecenoic acid, 1,2,3-propanetriyl ester, (E,E,E)	C_57_H_104_O_6_	1.50	Triglycerides
16	61.83	9-Octadecenoic Acid,	C_28_H_44_O_4_	1.83	Monounsaturated omega-9 fatty acid
17	64.47	Ethyl iso-allocholate	C_26_H_44_O_5_	1.63	Sterol lipids
18	64.90	Ethyl iso-allocholate	C_26_H_44_O_5_	1.92	Sterol lipids

**Table 4:** In silico docking results of *T. turbinata* oil nanoemulsion and the co-crystallized ligand against the targeted protein FASN (PDB: 7MHD).

Title	XP Gscore	Docking Score	Glide Emodel	Glide Gscore	MMGBSA dG Bind
Native ligand	−10.717	−10.717	−94.123	−10.717	−118.95
Octadecanoic acid, 4-hydroxy-, methyl ester	−9.084	−9.084	−52.752	−9.084	−107.55
Octanoic acid, 1-methyltridecyl ester	−7.845	−7.845	−51.711	−7.845	−93.02
Dodecanoic acid, 4-penten−1-yl ester	−7.377	−7.377	−52.066	−7.377	−106.07
9-Octadecenoic acid (Z) (oleic acid)	−7.117	−7.112	−44.634	−7.117	−85.17
Hexadecanoic acid, 1,5-pentanediyl ester	−6.86	−6.86	−51.479	−6.86	−121.79
Hexadecanoic acid (palmitic acid)	−6.207	−6.202	−40.19	−6.207	−91.7
Isosorbide	−6.021	−6.021	−27.854	−6.021	−42.4
Ethyl iso-allocholate (ethyl cholate)	−5.782	−5.782	−45.668	−5.782	−73.77
2-Hexenoic acid, 5-hydroxy−3,4,4-trimethyl	−4.78	−4.779	−20.008	−4.78	−21.6
Octanoic acid, oct−3-en−2-yl ester	−4.768	−4.768	−32.792	−4.768	−69.29
Dodecanoic acid (lauric acid)	−4.678	−4.673	−24.707	−4.678	−57.5

**Table 5:** In silico docking results of *T. turbinata* oil nanoemulsion and the co-crystallized ligand against the targeted protein KEAP1 (PDB: 4L7B).

Title	Docking Score	Glide Emodel	Glide Gscore	XP Gscore	MMGBSA dG Bind
Native ligand	−6.901	−65.747	−6.906	−6.906	−82.83
Octadecanoic acid, 4-hydroxy-, methyl ester	−5.876	−37.913	−5.876	−5.876	−78.13
Isosorbide	−5.476	−35.592	−5.476	−5.476	−41.83
2-Hexenoic acid, 5-hydroxy−3,4,4-trimethyl	−4.833	−22.565	−4.833	−4.833	−41.11
Ethyl iso-allocholate (ethyl cholate)	−4.223	−43.377	−4.223	−4.223	−82.28
Octanoic acid, 1-methyltridecyl ester	−3.761	−44.763	−3.761	−3.761	−72.83
Dodecanoic acid, 4-penten−1-yl ester	−3.239	−39.713	−3.239	−3.239	−71.93
Hexadecanoic acid, 1,5-pentanediyl ester	−2.896	−35.155	−2.896	−2.896	−72.82
Hexadecanoic acid (palmitic acid)	−2.612	−27.439	−2.616	−2.616	−64.38
9-Octadecenoic acid (Z) (oleic acid)	−2.534	−29.811	−2.539	−2.539	−52.76
Dodecanoic acid (lauric acid)	−2.397	−21.574	−2.402	−2.402	−56.67
Octanoic acid, oct−3-en−2-yl ester	−2.265	−33.895	−2.265	−2.265	−56.72

**Table 6:** In silico docking results of marine compounds and the co-crystallized ligand against the DNA gyrase (GyrB) (PDB: 3U2K).

Title	XP Gscore	Docking Score	Glide Emodel	Glide Gscore	MMGBSA dG Bind
Native ligand	−4.936	−4.936	−73.155	−4.936	−94.81
9-Octadecenoic acid (Z) (oleic acid)	−4.7	−4.695	−36.626	−4.7	−85.09
Isosorbide	−4.545	−4.545	−25.708	−4.545	−37.26
Octanoic acid, 1-methyltridecyl ester	−4.454	−4.454	−38.552	−4.454	−70.06
Ethyl iso-allocholate (ethyl cholate)	−4.367	−4.367	−41.355	−4.367	−75.4
Hexadecanoic acid (palmitic acid)	−3.869	−3.864	−26.234	−3.869	−67.06
Octadecanoic acid, 4-hydroxy-, methyl ester	−3.789	−3.789	−33.873	−3.789	−79.25
Dodecanoic acid, 4-penten−1-yl ester	−3.508	−3.508	−40.217	−3.508	−72.4
Octanoic acid, oct−3-en−2-yl ester	−2.871	−2.871	−34.312	−2.871	−60.39
2-Hexenoic acid, 5-hydroxy−3,4,4-trimethyl	−2.828	−2.827	−17.638	−2.828	−15.23
Hexadecanoic acid, 1,5-pentanediyl ester	−2.606	−2.606	−33.956	−2.606	−75.3
Dodecanoic acid (lauric acid)	−2.58	−2.575	−27.816	−2.58	−56.14
9-Octadecenoic acid, 1,2,3-propanetriyl ester, (E,E,E/Trielaidin)	−2.317	−2.317	−51.481	−2.317	−68.06

**Table 7:** Key QikProp-predicted properties for the three prioritized hits.

Property or Descriptor	Range or Recommended Values	Octadecanoic Acid, 4-Hydroxy-, methyl es	Isosorbide	2-Hexenoic Acid, 5-Hydroxy−3,4,4-trimeth	Ethyl Iso-Allocholate/Ethyl Cholate	Octanoic Acid, 1-Methyltridecyl Ester	Dodecanoic Acid, 4-Penten−1-yl Ester	Hexadecanoic Acid, 1,5-Pentanediyl Ester	Hexadecanoic Acid/Palmitic Acid	9-Octadecenoic Acid (Z) (Oleic Acid)	Dodecanoic Acid (Lauric Acid)	Octanoic Acid, Oct−3-en−2-yl ester	Dodecanoic Acid (Lauric Acid)
#Stars	0–5	4	6	0	1	6	2	3	3	2	3	1	3
#RtvFG	0–2	1	0	1	1	1	2	0	0	0	0	1	0
CNS	−2 (inactive), +2 (active)	−2	0	−1	−2	−1	−2	−2	−1	−2	−2	0	−2
Mol_MW	130.0–725.0	314.507	146.143	172.224	436.631	340.588	268.439	268.482	256.428	282.465	200.32	254.412	200.32
Dipole	1.0–12.5	1.214	1.289	2.449	6.449	2.478	3.688	3.565	2.918	3.281	2.635	2.056	2.635
SASA	300.0–1000.0	774.54	293.395	392.595	683.335	764.809	620.149	665.665	597.759	708.038	527.732	590.775	527.732
DonorHB	0.0–6.0	1	2	2	3	0	0	0	1	1	1	0	1
AcceptHB	2.0–20.0	3.7	6.8	3.7	7.1	2	4	2	2	2	2	2	2
QplogPo/w	−2.0–6.5	5.369	−0.813	1.265	3.653	7.253	4.046	5.428	5.057	5.849	3.667	4.925	3.667
QplogS	−6.5–0.5	−6.266	−0.364	−1.597	−4.741	−6.733	−3.595	−5.18	−4.151	−6.026	−3.52	−4.574	−3.52
QPlogHERG	concern below −5	−5.499	−1.713	−1.021	−3.698	−4.883	−4.182	−4.656	−2.414	−3.481	−2.416	−4.181	−2.416
QplogBB	−3.0–1.2	−1.658	−0.229	−0.769	−1.094	−0.872	−1.032	−1.185	−0.909	−1.459	−1.007	−0.332	−1.007
#Metab	1–8	2	4	2	4	1	3	1	1	3	1	3	1
QplogKhsa	−1.5–1.5	0.788	−0.885	−0.616	0.535	1.395	0.177	0.802	0.325	0.72	−0.004	0.626	−0.004
% Human Oral Absorption	>80% is high <25% is poor	100	76.983	71.609	100	100	100	100	93.312	92.05	92.142	100	92.142

Abbreviations: #Stars, number of ADMET property violations; #RtvFG, number of reactive functional groups; CNS, central nervous system (activity score); Mol_MW, Molecular weight; Dipole, computed dipole moment; SASA, total solvent accessible surface area; DonorHB, estimated number H+ to be donated in HB; AcceptHB, estimated number H+ to be accepted in HB; QplogPo/w: predicted octanol/water partition coefficient; QplogS: predicted aqueous solubility; QPlogHERG, predicted IC50 value for blockage of HERG K+ channels; QplogBB, predicted brain/blood partition coefficient; #Metab, number of possible metabolic reactions; QplogKhsa: prediction of binding to human serum albumin; % Human Oral Absorption, predicted human oral absorption on a 0 to 100% scale.

**Table 8:** Antibacterial activity of silver-loaded *T. turbinata* oil nanoemulsion compared to the antibiotic reference, measured by the clear zone (mm).

Microorganisms	Microorganisms	Control Antibiotic Gentamicin
*Bacillus subtilis* (*ATCC 6633*)	28 ± 1.0	25 ± 0.6
*Staph. aureus* (*ATCC 6538*)	20 ± 0.5	19 ± 0.8
*Pseudomonas aeruginosa* (*ATCC90274*)	25 ± 0.7	18 ± 0.2
*Salmonella typhi* (*ATCC 6539*)	18 ± 0.4	17 ± 0.1

**Table 9:** The MIC, MBC and the MBC/MIC Index of the Ag-algal oil nanoemulsion (Ag-TTO-NE) against *B. subtilis* (ATCC 6633) and *S. aureus* (ATCC 6538) Gram-positive bacteria and *P. aeruginosa* (*ATCC90274*) and *Salmonella typhi* (ATCC 6539).

Sample Code (1)	MIC (µg/mL)	MBC (µg/mL)	MBC/MIC Index
*Bacillus subtilis* (*ATCC 6633*)	15.62	31.25	2
*Staph. aureus* (*ATCC 6538*)	15.62	31.25	2
*Pseudomonas aeruginosa* (*ATCC90274*)	15.62	31.25	2
*Salmonella typhi* (*ATCC 6539*)	31.25	62.5	2

## Data Availability

Data will be made available on request.

## Supplementary Materials

The following supporting information can be downloaded at: https://www.mdpi.com/article/10.3390/md24070244/s1, Table S1. Full QikProp property output for the marine compounds.
