# Supplementary material for: Silver-Loaded Turbinaria turbinata Oil Nanoemulsions: Antimicrobial and Anticancer Potential Revealed Through In Vitro Assays and Molecular Docking
Source: Mar Drugs. 2026 Jul 13;24(7):244. doi: 10.3390/md24070244 (PMC13412699; doi:10.3390/md24070244)
Supplement: Supplementary file 1 [file marinedrugs-24-00244-s001.zip › marinedrugs-4394119-supplementary.pdf]

Silver-loaded *Turbinaria turbinata* oil nanoemulsions: Antimicrobial and Anticancer Potential Revealed through In Vitro Assays and Molecular Docking

Supplementary Information

Table S1. Full QikProp property output for the marine compounds.

Property or Descriptor	Range or recommended values	Octadecanoic acid, 4-hydroxy-, methyl es	Isosorbide	2-Hexenoic Acid, 5-Hydroxy-3,4,4-trimeth	Ethyl iso-allocholate / Ethyl cholate	Octanoic acid, 1-methyltridecyl ester	2,5-Heptadecadione	Hexadecanoic acid, 1,5-pentanediyl ester	Hexadecanoic Acid / palmitic acid	9-Octadecenoic acid (Z) (Oleic Acid)	Dodecanoic acid (lauric acid)	Octanoic acid, oct-3-en-2-yl ester	Dodecanoic acid (lauric acid)
**#stars**	0 – 5	4	6	0	1	6	2	3	3	2	3	1	3
**#amine**	0 – 1	0	0	0	0	0	0	0	0	0	0	0	0
**#amidine**	0	0	0	0	0	0	0	0	0	0	0	0	0
**#acid**	0 – 1	0	0	1	0	0	0	0	1	1	1	0	1
**#amide**	0 – 1	0	0	0	0	0	0	0	0	0	0	0	0
**#rotor**	0 – 15	17	2	4	8	18	14	16	14	15	10	11	10
**#rtvFG**	0 – 2	1	0	1	1	1	2	0	0	0	0	1	0
**CNS**	−2 (inactive), +2 (active)	-2	0	-1	-2	-1	-2	-2	-1	-2	-2	0	-2
**mol_MW**	130.0 – 725.0	314.507	146.143	172.224	436.631	340.588	268.439	268.482	256.428	282.465	200.32	254.412	200.32
**dipole†**	1.0 – 12.5	1.214	1.289	2.449	6.449	2.478	3.688	3.565	2.918	3.281	2.635	2.056	2.635
**SASA**	300.0 – 1000.0	774.54	293.395	392.595	683.335	764.809	620.149	665.665	597.759	708.038	527.732	590.775	527.732
**FOSA**	0.0 – 750.0	672.206	194.87	248.358	551.035	731.719	541.297	589.05	532.19	589.029	426.855	558.238	426.855
**FISA**	7.0 – 330.0	102.335	98.525	138.983	132.299	33.09	78.852	76.615	65.569	100.334	100.878	24.583	100.878

**PISA**	0.0 – 450.0	0	0	5.254	0	0	0	0	0	18.675	0	7.953	0
**WPSA**	0.0 – 175.0	0	0	0	0	0	0	0	0	0	0	0	0
**volume**	500.0 – 2000.0	1329.245	464.645	646.93	1353.864	1404.143	1110.4	1170.598	1046.183	1204.712	867.646	1036.972	867.646
**donorHB**	0.0 – 6.0	1	2	2	3	0	0	0	1	1	1	0	1
**accptHB**	2.0 – 20.0	3.7	6.8	3.7	7.1	2	4	2	2	2	2	2	2
**dip^2/V†**	0.0 – 0.13	0.001108	0.003577	0.009272	0.030722	0.004374	0.012249	0.010856	0.008137	0.008934	0.008003	0.004078	0.008003
**ACxDN^.5/SA**	0.0 – 0.05	0.004777	0.032777	0.013328	0.017996	0	0	0	0.003346	0.002825	0.00379	0	0.00379
**glob**	0.75 – 0.95	0.754845	0.988842	0.921423	0.866127	0.792902	0.836222	0.806952	0.833765	0.773328	0.833642	0.838664	0.833642
**QPpolrz**	13.0 – 70.0	36.127	11.157	17.218	42.891	38.482	29.295	30.42	26.725	32.605	22.148	28.358	22.148
**QPlogPC16**	4.0 – 18.0	11.765	4.44	5.929	12.348	11.567	9.084	9.637	8.6	10.565	7.067	8.06	7.067
**QPlogPoct‡**	8.0 – 35.0	14.065	9.49	10.06	22.538	12.395	10.812	9.933	10.136	12.17	8.804	9.123	8.804
**QPlogPw**	4.0 – 45.0	3.638	10.281	7.336	11.886	-0.216	2.599	0.283	1.95	2.358	2.904	0.906	2.904
**QPlogPo/w**	−2.0 – 6.5	5.369	-0.813	1.265	3.653	7.253	4.046	5.428	5.057	5.849	3.667	4.925	3.667
**QPlogS**	−6.5 – 0.5	-6.266	-0.364	-1.597	-4.741	-6.733	-3.595	-5.18	-4.151	-6.026	-3.52	-4.574	-3.52
**CIQPlogS**	−6.5 – 0.5	-4.118	-0.316	-1.348	-5.043	-5.378	-2.918	-3.847	-3.576	-4.111	-2.438	-3.548	-2.438
**QPlogHERG**	concern below −5	-5.499	-1.713	-1.021	-3.698	-4.883	-4.182	-4.656	-2.414	-3.481	-2.416	-4.181	-2.416
**QPPCaco**	<25 poor, >500 great	1060.378	1152.37	120.65	551.194	4809.565	1770.691	1859.337	599.392	280.564	277.254	5791.324	277.254

**QPlogBB**	−3.0 – 1.2	-1.658	-0.229	-0.769	-1.094	-0.872	-1.032	-1.185	-0.909	-1.459	-1.007	-0.332	-1.007
**QPPMDCK**	<25 poor, >500 great	527.072	576.665	63.978	259.855	2701.629	917.409	967.151	361.843	159.285	157.255	3302.346	157.255
**QPlogKp**	−8.0 – −1.0	-1.773	-3.143	-3.678	-3.19	-0.401	-1.629	-1.395	-1.384	-1.863	-2.419	-0.888	-2.419
**IP(ev)†**	7.9 – 10.5	11.11	10.635	10.158	10.426	10.832	10.704	10.68	10.927	9.823	10.926	9.947	10.926
**EA(eV)†**	−0.9 – 1.7	-0.917	-2.254	-0.295	-0.915	-1.037	-0.477	-0.691	-1.024	-1.014	-1.028	-0.873	-1.028
**#metab‡**	1 – 8	2	4	2	4	1	3	1	1	3	1	3	1
**QPlogKhsa**	−1.5 – 1.5	0.788	-0.885	-0.616	0.535	1.395	0.177	0.802	0.325	0.72	-0.004	0.626	-0.004
**Human Oral Absorption**	1, 2, or 3 for low, medium, or high.	1	3	3	3	1	3	1	3	3	3	3	3
**% Human Oral Absorption**	>80% is high; <25% is poor	100	76.983	71.609	100	100	100	100	93.312	92.05	92.142	100	92.142
**SAFluorine**	0.0 – 100.0	0	0	0	0	0	0	0	0	0	0	0	0
**SAamideO**	0.0 – 35.0	0	0	0	0	0	0	0	0	0	0	0	0
**PSA**	7.0 – 200.0	58.942	62.897	68.789	91.22	29.525	50.789	38.061	44.837	48.955	49.361	28.855	49.361
**#NandO**	2 – 15	3	4	3	5	2	2	1	2	2	2	2	2
**RuleOfFive**	maximum is 4	1	0	0	0	1	0	1	1	1	0	0	0
**RuleOfThree**	maximum is 3	0	8	0	17	0	0	0	0	0	0	0	0
**#ringatoms**	Number of atoms in a ring	0	0	0	0	0	0	0	0	0	0	0	0
**#in34**	Number of atoms in 3- or 4-membered rings	0	8	0	17	0	0	0	0	0	0	0	0

**#in56**	Number of atoms in 5- or 6-membered rings	0	6	0	17	0	0	0	0	0	0	0	0
**#noncon**		22	10	12	31	24	19	19	18	20	14	18	14
**#nonHatm**	Number of heavy atoms (nonhydrogen atoms)	1	0	0	0	1	0	0	0	1	0	0	0
**Jm**	Predicted maximum transdermal transport rate	0.003	45.429	0.913	0.005	0.025	1.603	0.071	0.748	0.004	0.231	0.876	0.231
